# Interplay between Graph Topology and Correlations of Third Order in Spiking Neuronal Networks

**DOI:** 10.1371/journal.pcbi.1004963

**Published:** 2016-06-06

**Authors:** Stojan Jovanović, Stefan Rotter

**Affiliations:** 1 Bernstein Center Freiburg & Faculty of Biology, University of Freiburg, Freiburg, Germany; 2 CB, CSC, KTH Royal Institute of Technology, Stockholm, Sweden; Indiana University, UNITED STATES

## Abstract

The study of processes evolving on networks has recently become a very popular research field, not only because of the rich mathematical theory that underpins it, but also because of its many possible applications, a number of them in the field of biology. Indeed, molecular signaling pathways, gene regulation, predator-prey interactions and the communication between neurons in the brain can be seen as examples of networks with complex dynamics. The properties of such dynamics depend largely on the topology of the underlying network graph. In this work, we want to answer the following question: Knowing network connectivity, what can be said about the level of third-order correlations that will characterize the network dynamics? We consider a linear point process as a model for pulse-coded, or spiking activity in a neuronal network. Using recent results from theory of such processes, we study third-order correlations between spike trains in such a system and explain which features of the network graph (i.e. which topological motifs) are responsible for their emergence. Comparing two different models of network topology—random networks of Erdős-Rényi type and networks with highly interconnected hubs—we find that, in random networks, the average measure of third-order correlations does not depend on the local connectivity properties, but rather on global parameters, such as the connection probability. This, however, ceases to be the case in networks with a geometric out-degree distribution, where topological specificities have a strong impact on average correlations.

## Author Summary

Many biological phenomena can be viewed as dynamical processes on a graph. Understanding coordinated activity of nodes in such a network is of some importance, as it helps to characterize the behavior of the complex system. Of course, the topology of a network plays a pivotal role in determining the level of coordination among its different vertices. In particular, correlations between triplets of events (here: action potentials generated by neurons) have recently garnered some interest in the theoretical neuroscience community. In this paper, we present a decomposition of an average measure of third-order coordinated activity of neurons in a spiking neuronal network in terms of the relevant topological motifs present in the underlying graph. We study different network topologies and show, in particular, that the presence of certain tree motifs in the synaptic connectivity graph greatly affects the strength of third-order correlations between spike trains of different neurons.

## Introduction

Analyzing networks of interacting elements has become the tool of choice in many areas of biology. In recent years, network models have been used to study the interactions between predator and prey [1], gene interactions [2] and neural network dynamics [3, 4]. A fundamental question in the study of complex networks is how the topology of the graph on which a dynamic process evolves influences its activity. A particularly interesting issue is the emergence of synchronized, or correlated patterns of events. While it is obvious that the presence or absence of such patterns of activity depends largely on how individual nodes in the network are connected, it is by no means a trivial task to explain exactly how this happens.

In theoretical neuroscience, the connection between network topology and correlated activity continues to be an important topic of study. Not only are correlations between neuronal spike trains believed to have an important function in information processing [5, 6] and coincidence detection [7], but they are also believed to be tied to expectation and attention (see [7] for details). In addition, it been shown that nerve cells can be extremely sensitive to synchronous input from large groups of neurons [8].

While there has been much work on elucidating the causes and effects of pairwise correlations between spike trains [3], it seems that correlations beyond second order also have a role to play in the brain. For example, it was indicated that a nonlinear neuron’s firing rate profile depends on higher-order correlations between the presynaptic spikes [9]. Higher-order correlations have also been reported in the rat somatosensory cortex and the visual cortex of the behaving macaque [10]. Indeed, it has been suggested that these correlations are inherent properties of cortical dynamics in many species [11, 12]. As a result, neural data has recently been intensively investigated for signs of higher-order synchrony using classical means such as maximum entropy models [13–18]. In addition, new methods are being developed in order to shed more light on what seems to be a very important property of networks in the brain [19–21].

In this work, we study the relation between the topology (i.e. synaptic connectivity) and correlations of third order between neuronal spike trains. Our aim was to show how triplet correlations depend on topological motifs in a network with known connectivity. We hope our results can be used to facilitate thought experiments to relate hypothetical connectivity to third-order correlations by, for example, assuming specific network topologies and then computing how these assumptions affect the dynamics.

In the following text, the word “connection” is meant to be translated as “synapse”. While this might be a point of contention, in previous work, it was clearly shown that that a mapping between synaptically coupled spiking networks (e.g. comprising LIF neurons) and statistical, point process models, such as Hawkes process exist, with exactly the same underlying connectivity [22]. In addition, it has been demonstrated that synaptic connectivity can be reconstructed from simulated spike trains with very high fidelity, provided the network has a connectivity which is not too dense and not too sparse [23]. On the basis of these two results, we feel enough confidence to claim that in the Hawkes process network models considered here “connections” in terms of coupling kernels can be safely identified with “synapses” in a spiking neuronal network.

However, we would also like to point out that knowing the true connectivity in an experimental setting is close to impossible. Indeed, the connectivity matrices, obtained by statistical inference methods applied to neural data are rarely more than a proxy for the actual “anatomical” connectivity. In other words, the existence of a statistical relationship between the firings of two neural cells (“correlation”) does generally not imply the existence of an actual synapse between them. In addition, the inference of connectivity from neural data is confounded by undersampling. One can typically only record from a tiny fraction of all neurons that constitute the network, while most of the population remains effectively hidden to the experimenter.

Similar work, pertaining to the influence of connectivity on correlations of second order has already been published [3, 24–26]. In it, the authors dissect the contribution of specific structural motifs to the emergence of pairwise correlations in a recurrent network of interconnected point processes, meant to represent neurons communicating via spikes. Interpreting known mathematical results [27] in an original fashion, they show how the influence of recurrent input can be disentangled to take into account not only effects of direct connections, but also indirect connectivity. However, no such result exists in the case of more complex patterns, stemming from correlations of higher order. With this paper, we aim to fill this gap.

Analogously to [3], we show that measures of third-order correlations (known in the statistical literature as “third-order joint cumulants”) are also heavily influenced by the presence of certain topological motifs in the network graph. We find that the motifs in question can be thought of representing “common input to triplets of neurons” and that, in graph theory terms, they represent rooted trees with three leaf nodes. Furthermore, we obtain an expansion of the joint third cumulants in terms of a sum of weights of all such subgraphs and show that, in a regular network (that is, a network with fixed in- and out-degrees), this expansion can be approximated by a formula that doesn’t depend on the specific adjacency matrix, but rather on global connectivity parameters, such as the connection probability *p*. In addition, our result extends to large random Erdős-Rényi type networks, as they are approximately regular when the number of nodes grows without bound. We find that the formula we derive is a useful approximation for quantifying the level of third-order correlations in networks with a narrow out-degree distribution. In addition, we look at networks of highly interconnected hubs and show that, in this case, the average joint third cumulant depends strongly on the details of the connectivity pattern.

## Methods

### The Hawkes process as a model of spiking neural networks

To study higher-order correlations in networks of spiking neurons with a fixed network topology, we apply a point process model introduced in [27, 28], which we will refer to as the “Hawkes process”. As the theory of Hawkes processes is rich and rather technical, we will only summarize the important definitions and equations needed to present our results. A more formal and thorough treatment of the model can be found in Hawkes’ original papers.

In what follows, we will use capital letters to denote matrices. Vectors will not be explicitly marked, as their identity will be clear from the context. Individual components of matrices and vectors are referred to by indices attached to the symbol. Furthermore, note that, from here onwards, the phrase “third-order correlations” should always be interpreted as referring to “third-order joint cumulants” (defined below).

Our spiking neuronal network consists of *N* neurons, of which *N*_*E*_ are excitatory and *N*_*I*_ are inhibitory. Spike trains of neuron *i*, $S_{i}\left(t\right)=\sum_{n}\delta \left(t-t_{n}^{i}\right)$, are modeled as realizations of point processes with time-dependent firing rates Λ_*i*_(*t*). In other words, we have

$$\Lambda_{i}\left(t\right)=E\left[S_{i}\left(t\right)|S_{j}\left(t^{\prime }\right),t^{\prime }\leq t,1\leq j\leq N\right],$$ (1)

where E[·] is the (conditional) expectation operator. In the Hawkes process framework, the vector Λ(*t*) of instantaneous firing rates (conditional on *S*_*i*_(*t*′), for *t*′ ≤ *t*) is given by

$$\Lambda \left(t\right)=\mu +\int_{-\infty }^{t}G\left(t-t^{\prime }\right)\cdot S\left(t^{\prime }\right)dt^{\prime }\equiv \mu +\left(G⋆S\right)\left(t\right).$$ (2)

The vector *μ* can be interpreted as the rate of spontaneous activity (due to constant external input) in the network. The neurons in the network would independently spike at rates, given by components of vector *μ*, if there were no synaptic connections between neurons in the network.

Recurrent synaptic interaction in the network is governed by the matrix of interaction kernels *G*(*t*), an *N* × *N* matrix of causal functions *g*_*ij*_(*t*), describing the influence of a spike in neuron *j* imposed on the future rate of neuron *i*. Typically, this is a sparse matrix with most entries being zero, and only few of them being nonzero. In principle, all of the functions *g*_*ij*_(*t*) can be different. However, for the sake of simplicity, we will assume that all source neurons in the excitatory subpopulation have interaction kernels equal to *g*_*E*_(*t*) to contact their targets, and all inhibitory neurons have interaction kernels *g*_*I*_(*t*). Thus, the total synaptic weight of excitatory neurons equals *g*_*E*_ ≡ ∫*g*_*E*_(*t*) *dt* and is positive, i.e. *g*_*E*_ > 0. Similarly, for inhibitory neurons, *g*_*I*_ ≡ ∫*g*_*I*_ (*t*) *dt* < 0.

The number *g*_*E*_ represents the expected number of extra spikes in the postsynaptic (target) neuron induced by a spike of the presynaptic (source) neuron. Analogously, for inhibitory neurons, the number *g*_*I*_ represents the expected reduction in the total number of spikes produced by the postsynaptic neuron.

The exact connectivity between neurons in the network is chosen randomly, according to various rules, as will be explained in the sections to follow.

**Fig 1:**
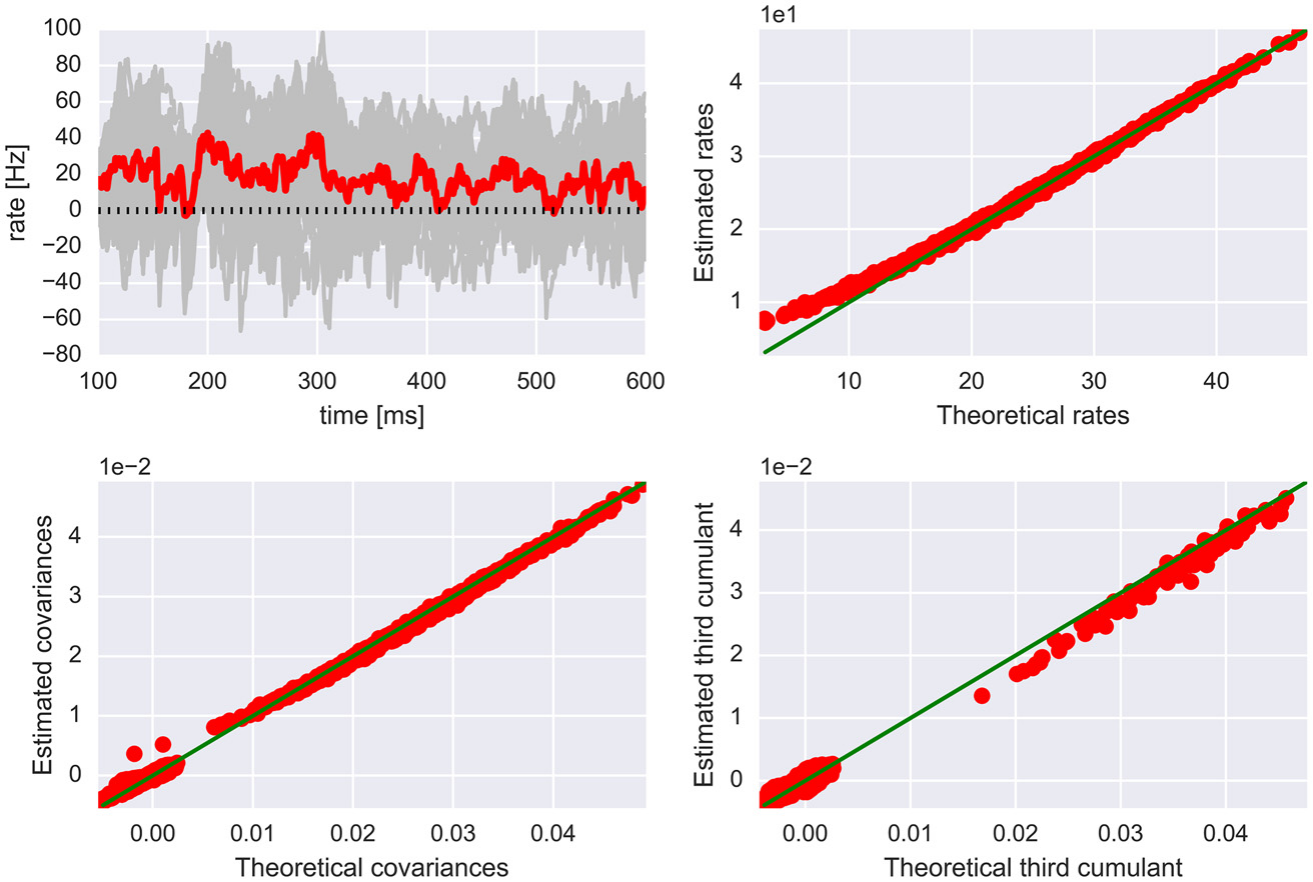
Hawkes process theory reproduces rates, correlations and third cumulants in a simulated network with Erdős-Rényi type random connectivity. Network parameters are *N* = 1000, *N*_*E*_ = 800, *N*_*I*_ = 200, *p* = 0.1, *g*_*E*_ = 0.015 and *g*_*I*_ = −0.075. Top left: Fluctuating firing rates of 50 randomly chosen neurons (gray traces) and their average (red line). The average rate only rarely goes below 0 (dashed line). Top right: Estimated temporal averages of firing rates scattered vs. rates predicted by Hawkes theory. The diagonal (green line) indicates a perfect match. Note that there is a slight discrepancy between theory and simulation for very low rates. Bottom left: Estimated integrated pairwise covariances (of all possible neuron pairs) scattered vs. integrated covariances predicted by Hawkes theory. Bottom right: Estimated integrated joint third cumulants (see the following sections for a definition) of a 100 randomly chosen neurons, scattered vs. integrated joint cumulants computed from Hawkes theory. The larger discrepancies are due to finite simulation time and a relatively small sample size.

One important thing to note is that the Hawkes model only allows for pairwise interactions, and yet possesses correlations of all orders. Furthermore, the Hawkes process is a probabilistic spike generator and, as such, may exhibit a different behavior than an encoder with a deterministic threshold mechanism. It is, however, important to realize that real neurons that are embedded in a large network possess both stochastic and deterministic features. Another potential limitation of the Hawkes model is that it provides a good approximation when synapses are weak, but strong synapses may more thoroughly explore neuronal nonlinearities. Finally, the Hawkes process is formally correctly defined only for positive interaction kernels. Negative interactions may lead to a rate vector Λ(*t*) with negative entries, which is of course not a meaningful configuration. Thus, technically, one should use the rectified rate [Λ(*t*)]_+_ as a basis for spike generation in simulations. In the following, we will assume that the probability of having negative entries in the rate vector is negligibly low and will ignore the rectifying non-linearity. The goodness of this approximation is illustrated in Fig 1.

At equilibrium, the expected firing rate vector of the Hawkes process, E[Λ(t)], no longer depends on time. We can compute the stationary rate vector, denoted Λ, as follows

$$\Lambda =\mu +\int_{-\infty }^{+\infty }\Lambda G\left(t-t^{\prime }\right)dt^{\prime }=\mu +\Lambda \left(\int_{-\infty }^{+\infty }G\left(t\right)dt\right),$$ (3)

from which we obtain the stationary rate of the network as

$$\Lambda ={\left(I-G\right)}^{-1}\mu ,$$ (4)

where we have used *G* as a shortcut for the matrix of integrated interaction kernels, i.e. *G* ≡ ∫*G*(*t*) *dt* and I denotes the *N* × *N* unit matrix. A summary of symbols, used in the text can be found in Table 1.

**Table 1:** Symbols used in text (in order of appearance).

Symbol	Description
*N*	total number of neurons
*N*_*E*_	number of excitatory neurons
*N*_*I*_	number of inhibitory neurons
*S*(*t*)	spike train vector
Λ(*t*)	conditional firing rate vector
*G*(*t*), *g*_*ij*_(*t*)	matrix of interaction kernels, its components
(*g*_*E*_) *g*_*E*_(*t*)	(integrated) excitatory neuron interaction kernel
(*g*_*I*_) *g*_*I*_(*t*)	(integrated) inhibitory neuron interaction kernel
*μ*, *μ*_*i*_	external input vector, external input to neuron *i*
Λ	stationary firing rate vector, Λ=E[Λ(t)]
*G*	integrated matrix of interaction kernels
*C*(*τ*)	covariance density matrix
*R*(*t*)	convolution power series of the matrix *G*(*t*)
*κ*^*ijk*^(*t*_1_, *t*_2_, *t*_3_)	third-order joint cumulant density of neurons *i*, *j* and *k*
*N*_*i*_(*T*)	spike count of neuron *i* in a bin of size *T*
*κ*^*ijk*^(*T*)	third cumulant of spike counts of neurons *i*, *j* and *k*
Ψ(*t*)	Ψ(t)=R(t)-Iδ(t)
*κ*^*ijk*^	integrated joint third cumulant of neurons *i*, *j* and *k*
*B*	power series of matrix *G*
*C*	integrated covariance density matrix
*N*_pop_(*T*)	population spike count in a bin of size *T*
${\bar{\kappa }}_{3}$	average joint third cumulant
*p*	connection probability
*μ*^(*k*)^	average common input, shared by *k* neurons
${\tilde{\kappa }}_{3}$	quadratic approximation of the average third cumulant ${\bar{\kappa }}_{3}$

In what follows, we will also restrict ourselves to systems in which the spectral radius of the matrix *G* (the largest eigenvalue of *G*), which we denote by *ρ*(*G*), is less than 1. Indeed, this condition insures the existence of the matrix inverse in the rate Eq 4. Furthermore, if *ρ*(*G*) > 1, it may happen that no stable equilibrium of the system exists and the spiking activity exhibits runaway solutions.

### Pairwise correlations in the Hawkes process framework

An important result, originally presented in Hawkes’ original work [27, 28], was that the lagged cross-covariance of spike trains of different neurons can be analytically computed directly from the matrix of interaction kernels *G*(*t*). More precisely, we can formally define the covariance density matrix, denoted by *C*(*τ*), as

$$C\left(\tau \right)=E\left[S\left(t+\tau \right)S{\left(t\right)}^{T}\right]-\Lambda \Lambda^{T}.$$ (5)

As was discussed before, intuitively, the entry (*i*, *j*) in *C*(*τ*) can be thought of as representing the probability that a spike of neuron *j* causes a spike of neuron *i* after time lag *τ*, minus the probability that this happens by chance (which, assuming stationarity, equals ΛΛ^*T*^). As noted in [27, 28], it is possible to rewrite *C*(*τ*) as

$$C\left(\tau \right)=D\delta \left(t\right)+C_{0}\left(\tau \right)-\Lambda \Lambda^{T},$$ (6)

where *D* ≡ diag(Λ) is a diagonal matrix, with the entries of the rate vector Λ on the diagonal. Furthermore, *C*_0_(*τ*) denotes the continuous part of the covariance density matrix, which is the solution to the matrix convolution equation

$$C_{0}\left(\tau \right)=G\left(\tau \right)D+\left(G⋆C_{0}\right)\left(\tau \right),\tau >0,$$ (7)

where the convolution of two matrix functions *F*(*t*) and *G*(*t*) equals a matrix function *H*(*t*) ≡ (*F* ⋆ *G*)(*t*) with

$$H_{ij}\left(t\right)=\int_{-\infty }^{t}F\left(t-s\right)\cdot G\left(s\right)ds=\sum_{k}\int_{-\infty }^{t}F_{ik}\left(t-s\right)G_{kj}\left(s\right)ds,$$ (8)

where ⋅ denotes the usual product of two numerical matrices. An important result in [28] is that the Fourier transform of the covariance density matrix, i.e. $\hat{C}\left(\omega \right)\equiv \int_{-\infty }^{+\infty }C\left(\tau \right)e^{-i\omega \tau }d\tau$ can be expressed in terms of the Fourier transform $\hat{G}\left(\omega \right)$ of the matrix of interaction kernels *G*(*t*). More precisely, we have

$$\hat{C}\left(\omega \right)={\left(I-\hat{G}\left(\omega \right)\right)}^{-1}D{\left(I-{\hat{G}}^{*}\left(\omega \right)\right)}^{-1},$$ (9)

where * denotes the conjugate transpose of a matrix.

Recently, it has been shown [29, 30] that, component-wise and in the time domain, the previous equation can be written as

$$C_{ij}\left(\tau \right)=\sum_{k=1}^{N}\Lambda_{k}\int_{-\infty }^{+\infty }R_{ik}\left(u\right)R_{jk}\left(u+\tau \right)\;du,$$ (10)

where Λ_*k*_ is the *k*-th component of the previously defined stationary rate vector, and the matrix *R*(*t*) is a function of *G*(*t*). Namely, we have that *R*(*t*) is a “convolution power series” of *G*(*t*) or, more precisely,

$$R\left(t\right)=\sum_{n\geq 0}G^{⋆n}\left(t\right).$$ (11)

Here, the matrix *G*^⋆*n*^(*t*) denotes the *n*-th convolution power of the interaction kernel *G*(*t*), defined recursively by

$$G^{⋆0}\left(t\right)=I\delta \left(t\right),$$ (12)

$$G^{⋆n}\left(t\right)=\int_{-\infty }^{t}G^{⋆(n-1)}\left(t-s\right)\cdot G\left(s\right)ds,\;n\geq 1,$$ (13)

where ⋅ again denotes a matrix product. We have the following heuristic interpretation of the matrix elements *R*_*ij*_(*t*):

$$R_{ij}\left(t\right)\;dt\equiv P\left\{\text{spike of neuron }j\text{ at }0\text{ causes neuron }i\text{ to spike at }t\right\}.$$ (14)

This heuristic offer an interesting interpretation of Eq 10. Indeed, we can see the product Λ_*k*_ *R*_*ik*_(*u*)*R*_*jk*_(*u* + *τ*)*du* as representing the probability that neuron *k*, spiking at its stationary rate Λ_*k*_, causes neuron *i* to spike at *u* and neuron *j* at *u* + *τ*. The covariance density *C*_*ij*_(*τ*) of neurons *i* and *j* at lag *τ* is then nothing more than this probability, summed over all possible spikes times of neuron *i* (hence the integral w.r.t. *u*) and over all possible “presynaptic” neurons *k*. Thus, *C*_*ij*_(*τ*) can be seen as a sum of all possible ways in which a neuron *k* can induce activity in neurons *i* and *j*, with spikes that are *τ* apart.

**Fig 2:**
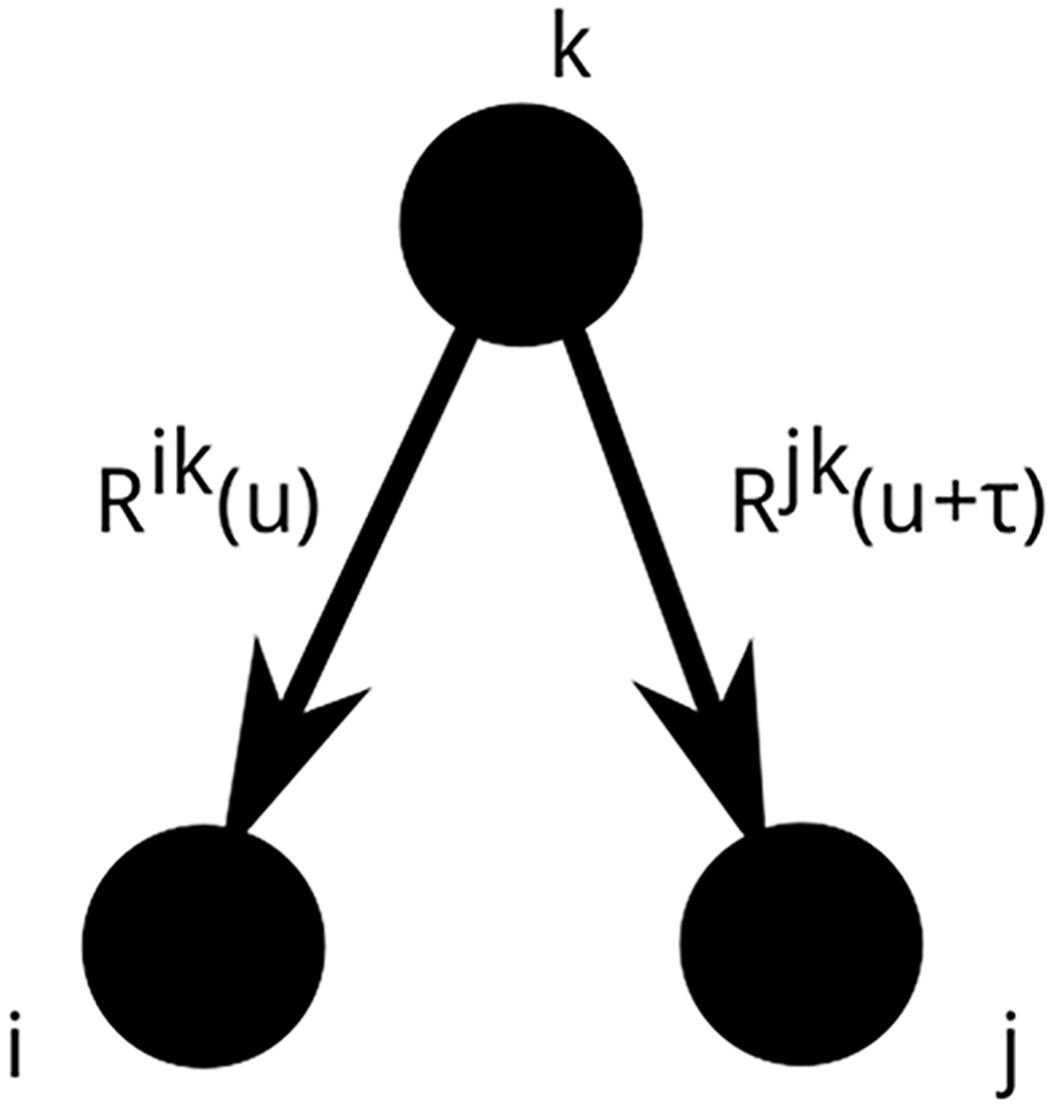
Pictorial representation of terms contributing to the pairwise covariance density. Each entry of *C*(*τ*) is a weighted sum of integral terms, corresponding to rooted trees with 2 leaves, *i* and *j*.

Moreover, a simple graphical representation of *C*_*ij*_(*τ*) is now available. As was first shown in [3], the product Λ_*k*_ *R*_*ik*_(*u*)*R*_*jk*_(*u* + *τ*) *du* can be represented as a rooted tree with leaves *i* and *j* (see Fig 2). Then, it can be shown that the lagged cross-covariance of spiking activity between neurons *i* and *j* is a sum of integral terms, each corresponding to a rooted tree with leaves *i* and *j* in the underlying network (for more details, see [3] and [29]).

### Higher order cumulants in analysis of network dynamics

We now move on to the problem of analyzing cumulants of higher order in networks of spiking neurons and introduce the tools necessary to do so. In statistics, a quantifier of third order correlations, analogous to the well-known covariance operator, is the third order joint cumulant, often denoted as *κ*_3_[*X*, *Y*, *Z*]. It measures above-chance level third order dependence in the same way that covariance does for second order. It is defined, for random variables *X*, *Y* and *Z*, as (see S3 Appendix. for a full derivation of the formula)

$$\kappa_{3}\left[X,Y,Z\right]=E\left[XYZ\right]-E\left[XY\right]E\left[Z\right]-E\left[XZ\right]E\left[Y\right]-E\left[YZ\right]E\left[X\right]+2E\left[X\right]E\left[Y\right]E\left[Z\right].$$ (15)

Let *i*, *j* and *k* be three distinct neurons in a recurrent neuronal network. Let further *A* = {(*i*, *t*_1_), (*j*, *t*_2_), (*k*, *t*_3_)} denote a spike pattern, where neuron *i* spikes at time *t*_1_, neuron *j* at *t*_2_ and neuron *k* at *t*_3_. If we now plug in the variables *S*_*i*_(*t*_1_), *S*_*j*_(*t*_2_) and *S*_*k*_(*t*_3_) into Eq 15 and denote

$$\kappa^{ijk}\left(t_{1},t_{2},t_{3}\right)\equiv \kappa_{3}\left[S_{i}\left(t_{1}\right),S_{j}\left(t_{2}\right),S_{k}\left(t_{3}\right)\right],$$ (16)

we see that the newly introduced function *κ*^*ijk*^(*t*_1_, *t*_2_, *t*_3_) measures the likelihood of the pattern *A* occurring not due to chance and not due to pairwise correlations.

Next, let *N*_*i*_(*T*) represent the number of spikes of neuron *i* in a time bin of size *T*. Then, clearly,

$$N_{i}\left(T\right)=\int_{0}^{T}S_{i}\left(t\right)\;dt.$$ (17)

Now, using Fubini’s theorem, we find that

$$\kappa^{ijk}\left(T\right)\equiv \kappa_{3}\left[N_{i}\left(T\right),N_{j}\left(T\right),N_{k}\left(T\right)\right]=\int_{0}^{T}\int_{0}^{T}\int_{0}^{T}\kappa^{ijk}\left(t_{1},t_{2},t_{3}\right)\;dt_{1}dt_{2}dt_{3}.$$ (18)

In other words, while the function *κ*^*ijk*^(*t*_1_, *t*_2_, *t*_3_) encodes the probability of occurrence of a single pattern *A*, the “integrated cumulant” *κ*^*ijk*^(*T*) (that is, the joint third cumulant of spike counts) measures the probability of the non-chance occurrence of any pattern of neurons *i*, *j* and *k* in a time bin of duration *T*. We will call the function *κ*^*ijk*^(*t*_1_, *t*_2_, *t*_3_) the *(3rd order) cumulant density*, as one needs to integrate it in order to obtain the 3rd cumulant of spike counts, i.e. *κ*^*ijk*^(*T*).

Assuming stationarity, the density *κ*^*ijk*^(*t*_1_, *t*_2_, *t*_3_) can be written (with slight abuse of notation) as a function of only the (two) time lags between spike events at *t*_1_, *t*_2_ and *t*_3_

$$\kappa^{ijk}\left(t_{1},t_{2},t_{3}\right)=\kappa^{ijk}\left(t_{2}-t_{1},t_{3}-t_{1}\right)\equiv \kappa^{ijk}\left(\tau_{1},\tau_{2}\right),$$ (19)

where we have defined *τ*_1_ = *t*_2_ − *t*_1_ and *τ*_2_ = *t*_3_ − *t*_1_. In that case, we get (see S1 Appendix)

$$\kappa^{ijk}\left(T\right)=\frac{\kappa_{3}\left[N_{i}\left(T\right),N_{j}\left(T\right),N_{k}\left(T\right)\right]}{T}=\int_{-T}^{T}\int_{-T}^{T}\kappa^{ijk}\left(\tau_{1},\tau_{2}\right)d\tau_{1}\;d\tau_{2}.$$ (20)

Thus, we obtain an alternative interpretation of *κ*^*ijk*^(*T*): It represents the third joint cumulant of spike counts of neurons *i*, *j* and *k* in a bin of size *T*, normalized by the bin size. As such, it is a quantity that can be easily computed from data, using unbiased estimators of higher-order cumulants, called *k*-statistics [31].

### Joint third cumulants in the Hawkes process framework

**Fig 3:**
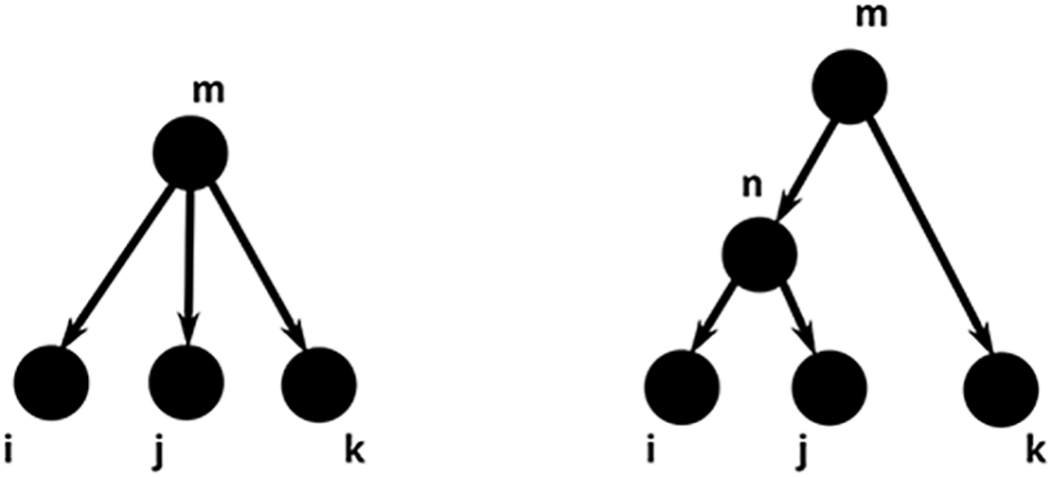
Pictorial representation of terms contributing to *κ*^ijk^(t_1_, t_2_, t_3_). Each *κ*^*ijk*^(*t*_1_, *t*_2_, *t*_3_) is a weighted sum of integral terms, corresponding to rooted trees with leaves *i*, *j* and *k* (see Eq 21). The first term maps to the left tree, while the three remaining terms correspond to three possible ways in which three labeled leaves can be arranged into two groups to form the tree on the right. The first group would represent the daughter nodes of vertex *m*, and the second group would be a single child of the root node *n*.

A recent result in the theory of Hawkes processes [29] shows that all 3rd order cumulant densities *κ*^*ijk*^(*t*_1_, *t*_2_, *t*_3_) can be computed, just as in the pairwise case, as sums of integral terms, each corresponding to a relevant topological motif (a subtree of the graph on which the process evolves), present in the underlying network. However, in the case of triplet correlations, the relevant rooted trees are somewhat more complicated (see Fig 3). Algebraically, we have

$$\begin{array}{l} \kappa^{ijk}(t_{1},t_{2},t_{3})=\overset{N}{\underset{m=1}{\sum }}\Lambda_{m}\int_{-\infty }^{+\infty }R_{im}(t_{1}-u)R_{jm}(t_{2}-u)R_{km}(t_{3}-u)du\\ \;+\overset{N}{\underset{m,n=1}{\sum }}\Lambda_{n}\int_{-\infty }^{+\infty }R_{in}(t_{1}-u)\left(\int_{-\infty }^{+\infty }R_{jm}(t_{2}-v)R_{km}(t_{3}-v)\Psi_{mn}(v-u)dv\right)du\\ \;+\overset{N}{\underset{m,n=1}{\sum }}\Lambda_{n}\int_{-\infty }^{+\infty }R_{jn}(t_{2}-u)\left(\int_{-\infty }^{+\infty }R_{im}(t_{1}-v)R_{km}(t_{3}-v)\Psi_{mn}(v-u)dv\right)du\\ \;+\overset{N}{\underset{m,n=1}{\sum }}\Lambda_{n}\int_{-\infty }^{+\infty }R_{kn}(t_{3}-u)\left(\int_{-\infty }^{+\infty }R_{im}(t_{1}-v)R_{jm}(t_{2}-v)\Psi_{mn}(v-u)dv\right)du, \end{array}$$ (21)

where Λ_*n*_ (the stationary rate of neuron *n*) and *R*_*ij*_(*t*) (the rate change at time *t* in neuron *i* caused by a spike of neuron *j* at 0) have been defined previously, and

$$\Psi \left(t\right)=R\left(t\right)-I\delta \left(t\right)=\sum_{n\geq 1}G^{⋆n}\left(t\right),$$ (22)

which, heuristically, simply means that

$$\Psi_{ij}\left(t\right)\;dt\equiv P\left\{\text{spike of neuron }j\text{ at }0\text{ causes neuron }i\;\neq \;j\text{ to spike at }t\;\neq \;0\right\}.$$ (23)

Unfortunately, this formula is cumbersome, impractical and difficult to work with. However, a much more elegant expression is obtained if one considers the previously defined joint cumulants of spike counts, *κ*^*ijk*^(*T*). Formally, considering infinitely large time bins

$$\kappa^{ijk}\equiv \lim_{T\to +\infty }\kappa^{ijk}\left(T\right)=\int_{-\infty }^{+\infty }\int_{-\infty }^{+\infty }\kappa^{ijk}\left(\tau_{1},\tau_{2}\right)\;d\tau_{1}d\tau_{2},$$ (24)

and letting $B\equiv {\left(I-G\right)}^{-1}$, where *G* is the previously defined integrated matrix of interaction kernels, we have [29]

$$\begin{array}{ll} \kappa^{ijk} & =\underset{m}{\sum }\Lambda_{m}B_{im}B_{jm}B_{km}\\ & +\underset{m,n}{\sum }\Lambda_{n}B_{im}B_{jm}(B_{mn}-\delta_{mn})B_{kn}\\ & +\underset{m,n}{\sum }\Lambda_{n}B_{jm}B_{km}(B_{mn}-\delta_{mn})B_{in}\\ & +\underset{m,n}{\sum }\Lambda_{n}B_{im}B_{km}(B_{mn}-\delta_{mn})B_{jn}. \end{array}$$ (25)

This can be considered as a generalization of the pairwise correlation result from [3]. Indeed, if we let *ω* = 0 in Eq 9 and set $C\equiv \hat{C}\left(0\right)=\int C\left(\tau \right)\;d\tau$, we have

$$C_{ij}=BDB^{*}=\sum_{m=1}^{N}\Lambda_{m}B_{im}B_{jm}.$$ (26)

The problem, of course, is that the collection of all integrated cumulants {*κ*^*ijk*^}_*i*, *j*, *k*_ represents a three-dimensional tensor, and as such cannot be represented in terms of a common matrix multiplication. For this reason, we must express *κ*^*ijk*^ as weighted sums and double sums of entries of the matrix *B* in formula 25.

### Populations cumulants as sums of joint cumulants of spike counts

Finally, let us touch upon the link between integrated covariances *C*_*ij*_, cumulants *κ*^*ijk*^, and moments of the population count distribution *N*_pop_(*T*) which we define as the sum of activity of all neurons in the network

$$N_{\text{pop}}\left(T\right)\equiv \sum_{m=1}^{N}N_{m}\left(T\right).$$ (27)

From the general properties of cumulants [32], one can prove that

$$\lim_{T\to +\infty }\frac{\text{Var}[N_{\text{pop}}\left(T\right)]}{T}=\sum_{i,j}C_{ij}.$$ (28)

In other words, the variance of the population activity is equal to the sum of all integrated covariances, normalized by bin size. Of course, this is only strictly true for infinitely large time bins, but we have found that Eq 28 is still a very good approximation whenever the size of bin *T* is much bigger than the temporal width of any entry in the matrix of interaction kernels *G*(*t*).

Likewise, one can prove that

$$\lim_{T\to +\infty }\frac{\kappa_{3}\left[N_{\text{pop}}\left(T\right)\right]}{T}=\sum_{i,j,k}\kappa^{ijk}.$$ (29)

Thus, the sums of all integrated cumulants of order 3 is equal to the third cumulant of population activity, normalized by bin size [31]. To understand why it is important to know the third cumulant *κ*_3_[*N*_pop_(*T*)] consider that, for a normally distributed random variable *X*, all cumulants of order 3 and higher are zero

$$X∼N\left(0,1\right)\Rightarrow \kappa_{n}\left[X\right]=0,\text{ for all }n\;\geq \;3.$$ (30)

Therefore, in a sense, non-zero cumulants of order 3 and higher measure the departure from normality of the variable *N*_pop_(*T*). Furthermore, in statistics, a measure of skewness of the distribution of a random variable *X* is defined as the (scaled) third cumulant *κ*_3_[*X*]. As the Gaussian distribution is symmetric about 0 (and thus *κ*_3_[*X*] = 0), any significant deviation of *κ*_3_[*N*_pop_(*T*)] indicates right (negative) or left (positive) skewness.

### Simulation and data analysis details

The simulation of linearly interacting point processes was conducted using the NEST simulator [33]. We simulated a network of 1000 neurons, of which 800 were excitatory and 200 inhibitory. The spikes of each neuron were generated according to a time-dependent rate function Λ(*t*), defined by Eq 2. Negative values of Λ(*t*) were rectified to zero, resulting in no spike output. Neurons received external Poissonian drive with constant rate of 10 Hz. Incoming spikes induced an increment of amplitude 1.5 Hz and −7.5 Hz for excitatory and inhibitory spikes, respectively, which decayed with a time constant of 10 ms. In the Hawkes process framework, this corresponds to an exponential interaction kernel with total integral *g*_*E*_ = 0.015 and *g*_*I*_ = −0.075, respectively. The synaptic delay was set to 2 ms. The simulation time step was 0.1 ms. The total simulation time was 5000 s = 5 ⋅ 10^6^ ms.

Spike data from simulations were sampled in time bins of duration *T* = 100 ms, producing 5 ⋅ 10^4^ bins. We found that the theoretical results concerning infinite sized bins are still largely valid when the bin size *T* is at least one order of magnitude larger than the interaction kernel time constant. The rates, pairwise covariances and joint third cumulants were estimated from a data matrix with 1000 rows (representing individual neurons) and 50000 columns (representing time bins) using *k*-statistics [31], which are known to be unbiased estimators of cumulants of any order. Note that pairwise covariances are nothing more that joint cumulants of second order.

## Results

### Weights of subtrees in the network determine the strength of triplet correlations

In this section we explain how recurrent connectivity affects joint third cumulants of triplets of neurons in a spiking neuronal network. As was mentioned before, the matrix of integrated interaction kernels *G* can be interpreted as an effective connectivity matrix, as each entry (*i*, *j*) represents the excess number of spikes in neuron *i*, caused by an individual spike in neuron *j*. With this in mind, let us now take a moment to develop a topological interpretation of Eq 25. Firstly, as *ρ*(*G*)<1 has been assumed, we have a power series expansion for the matrix $B={\left(I-G\right)}^{-1}$, namely *B* = ∑_*n*_ *G*^*n*^. In order to develop intuition, we first consider what happens to Eq 26 when we plug the power series expansion of *B* into it (as was done in [3]). The formula for *C*_*ij*_ reads

$$C_{ij}=\sum_{m=1}^{N}\sum_{r=0}^{+\infty }\sum_{s=0}^{+\infty }\Lambda_{m}G_{im}^{r}G_{jm}^{s}.$$ (31)

We now interpret the matrix *G*^*r*^ in the sense of graph theory, i.e. as a matrix whose entry (*i*, *j*) corresponds to the sum of compound weights of all paths from node *j* to node *i* in exactly *r* steps. Indeed, a typical entry of matrix *G*^*r*^ equals

$$G_{ij}^{r}=\sum_{k_{1},k_{2},\cdots ,k_{r-1}}G_{ik_{1}}G_{k_{1}k_{2}}\cdots G_{k_{r-1}j}.$$ (32)

We observe that each of the summands in the above equation is the average number of excess spikes, caused by an individual length *r* chain of spiking events, originating in neuron *j*. The entry $G_{ij}^{r}$ is then the sum over all such chains, i.e. over all possible intermediary neurons *k*_1_, *k*_2_, ⋯*k*_*r*−1_. Thus, a procedure for computing *C*_*ij*_ would go as follows:

1. Pick a “root neuron” *m*
2. Create a “spiking chain” from neuron *m* to neuron *i* that is *r* synaptic steps long
3. Create a “spiking chain” from neuron *m* to neuron *j* that is *s* neurons long
4. Compute the weight of the subtree defined in this way by multiplying together the weights of the branches (given by $G_{im}^{r}$ and $G_{jm}^{s}$)
5. Multiply everything by the “weight of the root node”, which we can formally define to be Λ_*m*_

Note that *r* = 0 (*s* = 0) is a distinct possibility (as the first term in the power series expansion of *B* is *G*^0^ ≡ *I*). In that case, we identify neurons *m* and *i* (*m* and *j*) and our “two-pronged tree” becomes a single branch with neuron *i* (*j*) on top and neuron *j* (*i*) on the bottom.

Our previous discussion shows that the integrated covariance density *C*_*ij*_ can be equivalently expressed as

$$C_{ij}=\sum_{T\in T_{ij}^{m}}w\left(T\right),$$ (33)

where the sum goes over the set $T_{ij}^{m}$ of all rooted trees *T* with root *m*, containing nodes *i* and *j*. Here, *w*(*T*) denotes the weight of tree *T*, defined as the product of weights of all edges, contained in *T*, times the weight of the root *m*, defined as being equal to Λ_*m*_.

Now, since, in the stationary case (see S1 Appendix)

$$C_{ij}=\lim_{T\to +\infty }\frac{\text{cov}[N_{i}\left(T\right),N_{j}\left(T\right)]}{T},$$ (34)

we have that, for infinitely large time bins, the probability (normalized by bin size) of the non-chance occurrence of ANY pattern of neurons *i* and *j* in a bin of size *T* can simply be computed as the sum of weights of ALL possible rooted trees with leaves *i* and *j*. Thus, in a nutshell, the only way pairwise interaction can arise between neurons *i* and *j* is through shared input by a neuron *k*, that can be arbitrarily far upstream from both *i* and *j*. This is the main result of [3].

With our intuition primed by consideration of the simpler, pairwise correlation case, we are ready to tackle the computation of *κ*^*ijk*^. Once again, plugging the power series expansion of matrix *B* into Eq 25 yields

$$\kappa^{ijk}=\sum_{T\in T_{ijk}^{m}}w\left(T\right),$$ (35)

where $T_{ijk}^{m}$ is the set of all rooted trees with root *m* containing nodes *i*, *j*, *k*, and *w*(⋅) is the already defined weight function. As we have that (see S1 Appendix)

$$\kappa^{ijk}=\lim_{T\to +\infty }\frac{\kappa_{3}\left[N_{i}\left(T\right),N_{j}\left(T\right),N_{k}\left(T\right)\right]}{T},$$ (36)

the interpretation of the “sum over trees” formula is analogous. In other words, for infinitely large time bins, the probability (normalized by bin size) of the non-chance occurrence of ANY pattern of neurons *i*, *j* and *k* in a bin of size *T* can simply be computed as the sum of weights of ALL possible rooted trees, containing nodes *i*, *j* and *k*. The only difference from the pairwise correlation case is that the topological motifs contributing to triplet correlations are different and more numerous.

**Fig 4:**
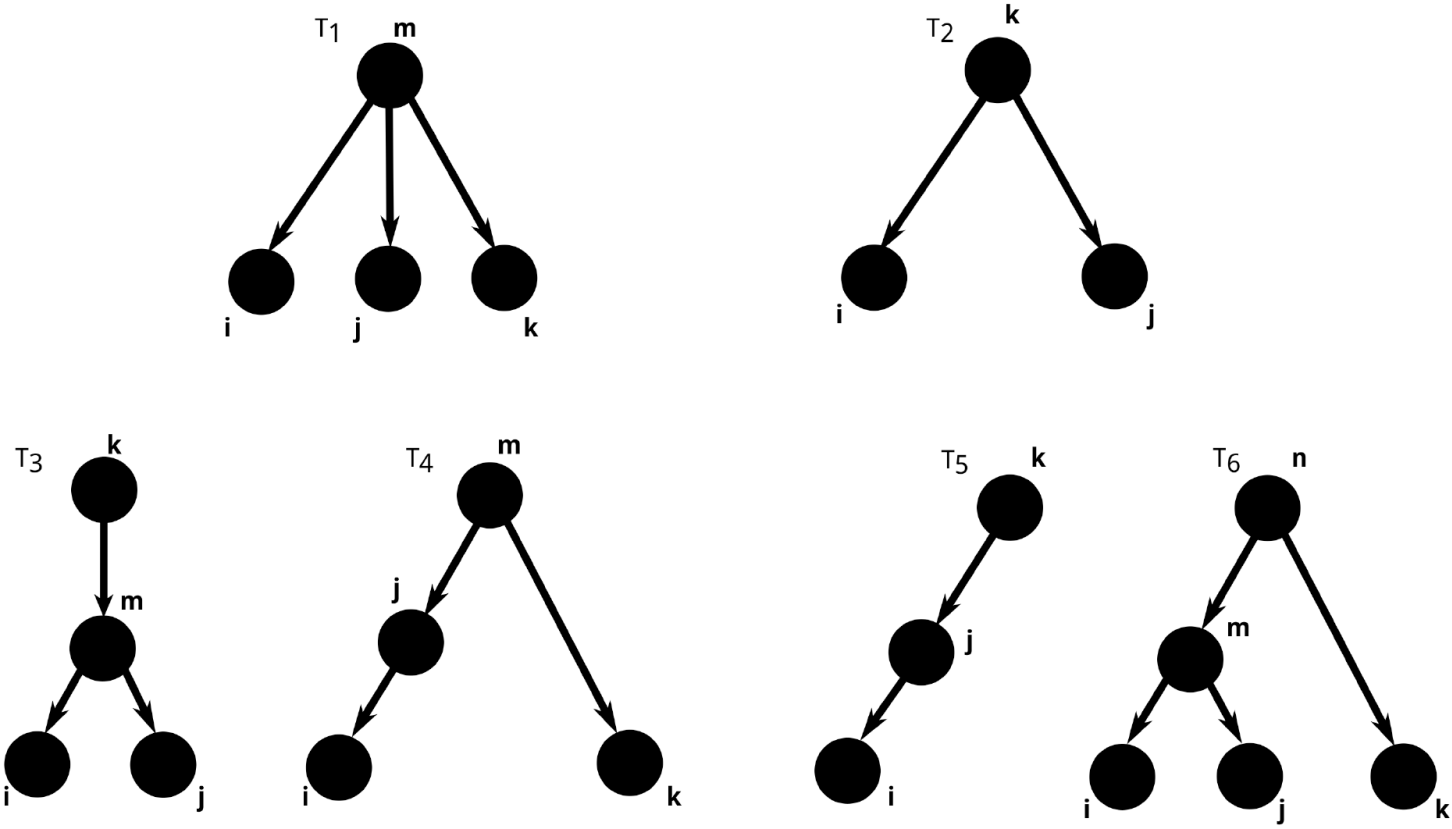
The pictorial representation of all terms, contributing to *κ*^ijk^. The tree shapes depicted in this figure were obtained from those in Fig 3 by performing all possible contractions of branches (see text).

What are the subtrees, contributing to *κ*^*ijk*^? We can get our first hint by comparing the formula 25 and the trees in Fig 3. Indeed, the first term in Eq 25 corresponds to the left, “three-pronged” tree in Fig 3—in fact, it is the combined weight of all such structures found in the graph with adjacency matrix *G*, summed over all possible identities of the root node *m* and over all possible lengths of the tree branches terminating at *i*, *j* and *k*. However, as any of the three branches can also be of length 0, the left tree in Fig 3 actually represents 4 different contributions to *κ*^*ijk*^, one corresponding to the tree depicted, in which case all of the branches are of length at least 1, and three other “two-pronged” trees obtained by collapsing one of the three branches and identifying the node *m* with node *i*, *j* or *k* (see first row of Fig 4). Algebraically, this can also be seen by replacing one of the *B* matrices in the first row of formula 25 by the identity matrix I. Indeed, placing I instead of *B* in any of the tree slots yields three possible contractions.

In the right tree in Fig 3, each of the last three terms in Eq 25 corresponds to one copy of it, the only difference among them being the label of the rightmost node. Indeed, the second term represents a tree in which the rightmost node is labeled *k*, for the third term the rightmost node is *i*, and for the last one it is *j*. Each of these terms contains three *B* matrices, and thus, each of these three terms will yield three additional trees whose weight will contribute to the overall sum, defining *κ*^*ijk*^ (see the second row of Fig 4). Like before, all of these are obtained by replacing one of the *B* matrices with the identity matrix I and performing the corresponding summation.

Notice that the last three terms in Eq 25 also depend on entries of the matrix B-I. This signifies the fact that the link between nodes *n* and *m* in Fig 3 can only “telescope out”, i.e. it cannot be contracted to 0 (indeed, it corresponds to the power series ∑_*n* ≥ 1_ *G*^*n*^ in which the term of order 0 is not present). For reasons as to why this branch does not allow contractions, see [29].

To summarize, the six different tree shapes depicted in Fig 4 all contribute terms that, when summed up, yield *κ*^*ijk*^. Likewise, as was mentioned previously, each branch, incident to each of the trees pictured, can have arbitrarily many intermediate nodes in between the two vertices shown.

### Average third cumulants in large random networks depend on the presence of a particular subtree

We are interested in computing the average third cumulant in the network, defined as

$$\frac{1}{N^{3}}\sum_{i,j,k}\kappa^{ijk},$$ (37)

where *κ*^*ijk*^ represent the integrated joint third cumulants of neurons *i*, *j* and *k*, considered previously. From Eq 29, we have that the previous sum equals

$$\lim_{T\to +\infty }\frac{\kappa_{3}\left[N_{\text{pop}}\left(T\right)\right]}{TN^{3}},$$ (38)

the third cumulant of population activity for an infinitely large time bin *T*, normalized by network size and bin width.

Note that the sum in Eq 37 goes over ALL indices *i*, *j* and *k*. Thus, we have three distinct cases:

1. The three indices are all distinct (*i* ≠ *j*, *j* ≠ *k*, *k* ≠ *i*);
2. the three indices are all equal (*i* = *j* = *k*);
3. two of three indices are equal, with the third being distinct (*i* = *j* and *j* ≠ *k*, or a permutation thereof).

The number of summands in the first case is equal to *N*(*N* − 1)(*N* − 2), in the second case it is simply *N*, and in the third one it equals 3*N*(*N* − 1). Thus, we have

$${\bar{\kappa }}_{3}=\frac{1}{N^{3}}\left[\sum_{i,j,k}\kappa^{ijk}+\sum_{i,j}\kappa^{iij}+\sum_{i}\kappa^{iii}\right].$$ (39)

In the limit of large networks, the first term becomes dominant, as

$$\lim_{N\to +\infty }\frac{3N(N-1)}{N^{3}}=0,\lim_{N\to +\infty }\frac{N}{N^{3}}=0,\;\text{but}\lim_{N\to +\infty }\frac{N(N-1)(N-2)}{N^{3}}=1.$$ (40)

Therefore, in all calculations that follow, we will assume that *i*, *j* and *k* are all different

$${\bar{\kappa }}_{3}=\frac{1}{N^{3}}\sum_{i\neq j\neq k}\kappa^{ijk}.$$ (41)

Furthermore, we assume the following about the underlying network topology:

1. (Random network condition) Every node *j* has probability *p* of forming a connection with any of the other *N* − 1 nodes.
2. (Generalized Dale’s law) To each node *j* we assign a *type* *l* ∈ *L* such that, for a fixed *j* we have ∀*i*, *g*_*ij*_ = *g*_*l*_.

In other words, the probability of a directed connection between any pair of nodes is equal to *p*, and each node is of a *single type* *l* and as such, only makes outgoing connections of type *l*. Here, *L* denotes the set of *type labels*.

The derivations that follow can still be done under these general assumptions. Also, note that, even though the first assumption allows for random topologies, the results obtained in this section hold true for regular networks as well, as very large random networks are approximately regular. However, in the interest of concreteness, we will assume that *L* = {*E*, *I*}. In short, each node *j* can either be of type *E* (excitatory) or type *I* (inhibitory). Thus, for a given “excitatory” node *j*, *g*_*ij*_ is either 0 (with probability 1 − *p*) or *g*_*E*_ (with probability *p*), for every neuron *i*. Likewise if the neuron is inhibitory (in that case, *g*_*ij*_ equals *g*_*I*_).

We now compute the average input to a neuron, embedded in the network. First, we note that, mathematically, the total input to node *i* can be computed as ∑_*j*_ *G*_*ij*_. Given our previous considerations, we have that the total input equals

$$p\left(N_{E}g_{E}+N_{I}g_{I}\right)=Np\left(\frac{N_{E}}{N}g_{E}+\frac{N_{I}}{N}g_{I}\right)\equiv N\mu^{in},$$ (42)

where *N*_*E*_ and *N*_*I*_ are the numbers of excitatory and inhibitory neurons in the network, respectively. We have also *μ*^*in*^ as $p(\frac{N_{E}}{N}g_{E}+\frac{N_{I}}{N}g_{I})$, the average strength of the total input to a neuron. Now, if we set the external input *μ* to 1, the stationary rate of neuron *i* can be seen to equal

$$\Lambda_{i}=\sum_{j}{\left[\sum_{n}G^{n}\right]}_{ij}=\sum_{j}\left(\delta_{ij}+G_{ij}+G_{ij}^{2}+\cdots \right)=\frac{1}{1-N\mu^{in}}\equiv \bar{\Lambda }.$$ (43)

Unsurprisingly, since the external input to all neurons is the same, the stationary rates are all equal ($\Lambda_{i}=\bar{\Lambda },\forall i$). The computation of the average cumulant ${\bar{\kappa }}_{3}$ can be done in much the same way (for details, see S2 Appendix). Note that, to simplify derivation, we assume that all neurons (irrespective of their type) have the same in-degree and out-degree. The final formula then reads

$$\begin{array}{ll} {\bar{\kappa }}_{3} & =-\frac{\bar{\Lambda }}{N^{3}}\frac{N^{4}p^{2}\mu^{\left(3\right)}}{{\left(1-\mu^{\left(1\right)}N\right)}^{3}}+\frac{3\bar{\Lambda }}{N^{3}}\frac{N^{3}p\mu^{\left(2\right)}}{{\left(1-\mu^{\left(1\right)}N\right)}^{2}}-\frac{3\bar{\Lambda }}{N^{3}}\frac{N^{4}p\mu^{\left(1\right)}\mu^{\left(2\right)}}{{\left(1-\mu^{\left(1\right)}N\right)}^{3}}\\ & -\frac{6\bar{\Lambda }}{N^{3}}\frac{N^{4}p\mu^{\left(1\right)}\mu^{\left(2\right)}}{{\left(1-\mu^{\left(1\right)}N\right)}^{3}}+\frac{6\bar{\Lambda }}{N^{3}}\frac{N^{3}{\left[\mu^{\left(1\right)}\right]}^{2}}{{\left(1-\mu^{\left(1\right)}N\right)}^{2}}+\frac{3\bar{\Lambda }}{N^{3}}\frac{N^{5}p^{3}{\left[\mu^{\left(2\right)}\right]}^{2}}{{\left(1-\mu^{\left(1\right)}N\right)}^{4}}, \end{array}$$ (44)

where each term in the equation corresponds to one of the tree shapes in Fig 4. We have chosen not to perform any simplifications in the formula, as we feel that this would obscure the correspondence each term has to its tree counterpart. Here, we have defined *μ*^(*k*)^ as the average common input, shared by *k* neurons, equaling

$$\mu^{\left(k\right)}=p\left(\frac{N_{E}}{N}g_{E}^{k}+\frac{N_{I}}{N}g_{I}^{k}\right).$$ (45)

Note that in this formalism, *μ*^(1)^ is the “average common input shared by one neuron”, equal to *μ*^*in*^, the average total input to a neuron. The precise nature of this relation between formula 44 and the topology of specific trees is covered in S2 Appendix. However, heuristically, the relationship is as follows

- The exponent of *N* in the formula counts the number of nodes of a particular tree
- The exponent of *p* is one less than the number of leaves of the tree
- The *μ*^(⋅)^ terms each correspond to an internal node of the tree (that is, a node that is not a leaf). The number in parenthesis in the superscript denotes the out-degree of that particular internal node. Thus, for example, *μ*^(3)^ indicates that the particular tree has an internal node with out-degree 3.
- The power *k* of the normalization factor $\frac{1}{{\left(1-\mu^{\left(1\right)}N\right)}^{k}}$ encodes the number of edges in the tree

Eq 44 can be used as an approximation whenever the degree distribution of the network in question is narrow–formally, it is only exactly true for a regular network, in which all neuron have the same in- and out-degrees. For large random networks of the Erdős-Rényi type, this is true as the resulting Binomial distributions have a standard deviation that vanishes with increasing network size. The numerical efficacy of such an approximation can be found in the following section.

A final thing to note about Eq 44 is what happens when *N* → +∞. Firstly, note that, once we perform all possible cancellations of terms in eq 44, we find, after rearranging

$${\bar{\kappa }}_{3}=3p^{3}{\left[\mu^{\left(2\right)}\right]}^{2}N^{2}{\bar{\Lambda }}^{5}-\left(9p\mu^{\left(1\right)}\mu^{\left(2\right)}+p^{2}\mu^{\left(3\right)}\right)N{\bar{\Lambda }}^{4}+\left(3p\mu^{\left(2\right)}+6{\left[\mu^{\left(1\right)}\right]}^{2}\right){\bar{\Lambda }}^{3}.$$ (46)

Thus, in the limit of large networks, the most important term is the one corresponding to tree *T*_6_ in Fig 4

$${\tilde{\kappa }}_{3}\equiv \frac{3\bar{\Lambda }}{N^{3}}\frac{N^{5}p^{3}{\left[\mu^{\left(2\right)}\right]}^{2}}{{\left(1-\mu^{\left(1\right)}N\right)}^{4}}=3p^{3}{\left[\mu^{\left(2\right)}\right]}^{2}N^{2}{\bar{\Lambda }}^{5},$$ (47)

since we have $1/\left(1-\mu^{\left(1\right)}N\right)=\bar{\Lambda }$. More precisely, we obtain the relation

$${\bar{\kappa }}_{3}={\tilde{\kappa }}_{3}+O\left(N\right)+O\left(1\right).$$ (48)

As is now evident, the contributions from all trees of this shape to ${\bar{\kappa }}_{3}$ grows as a quadratic function of *N*. The reason for this is that, in large networks, the number of “more complicated” subgraphs grows faster than the number of simpler ones. To see why this is true, consider counting all possible trees with *k* nodes and *k* − 1 edges in a random graph. Since each edge is generated independently, the number of such trees equals

$$\left(\begin{array}{c} N\\ k \end{array}\right)p^{k-1}{\left(1-p\right)}^{\left(\begin{array}{c} k\\ 2 \end{array}\right)-k+1},$$ (49)

Thus, as long as *k* ≤ ⌊*N*/2⌋, the number of tree structures with *k* nodes in a random graph of size *N* will increase with increasing *k*. This is, in a nutshell, why the most relevant contribution to ${\bar{\kappa }}_{3}$ comes from the “most complicated” tree, i.e. *T*_6_.

**Fig 5:**
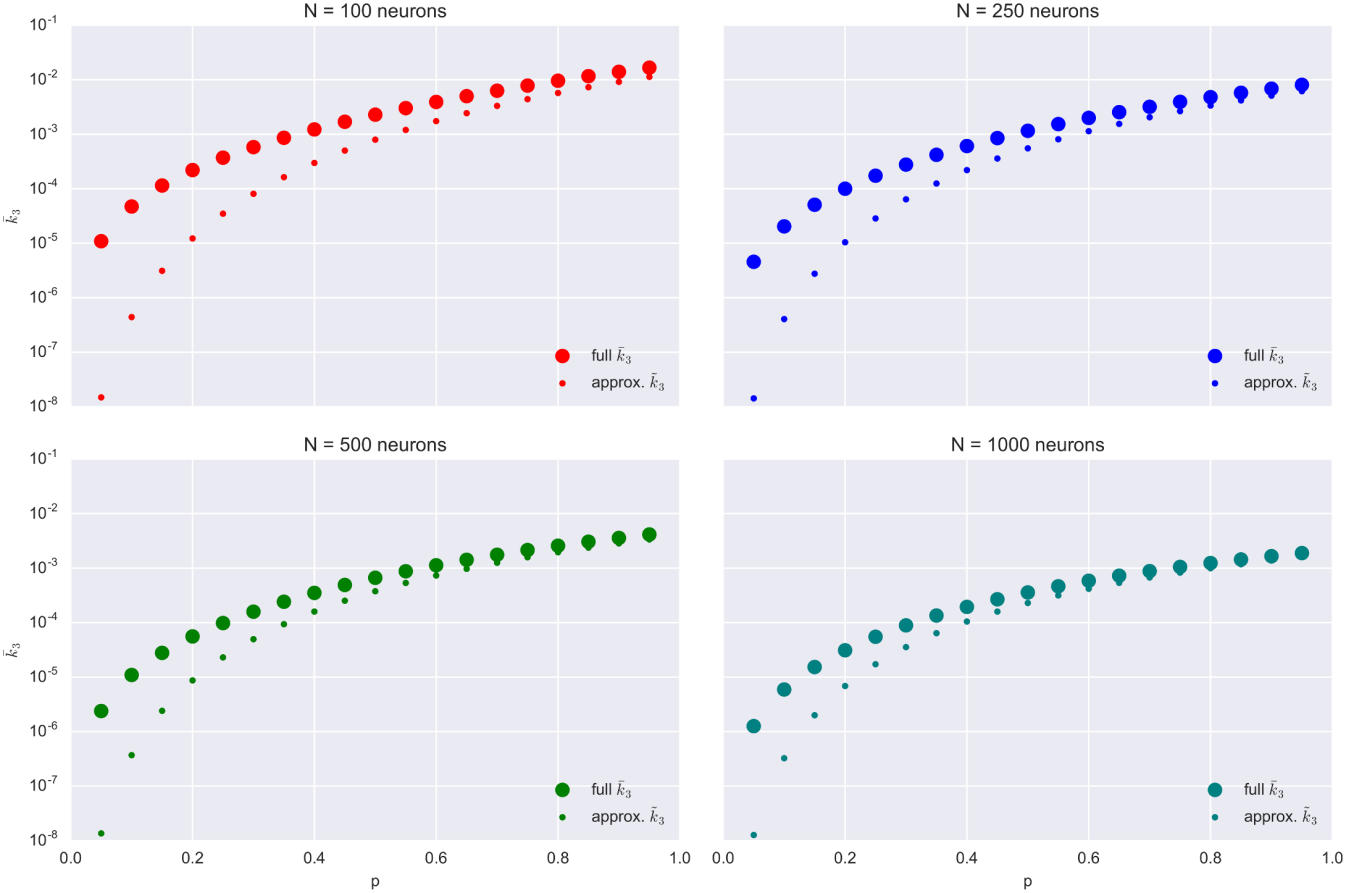
Efficacy of the quadratic approximation to ${\bar{\kappa }}_{3}$ (Eq 47) for different network sizes *N*. All four panels: ${\bar{\kappa }}_{3}$ and its quadratic approximation, ${\tilde{\kappa }}_{3}$, plotted for different values of the connection probability *p*. Each panel corresponds to a network of a given size.

With the previous discussion in mind, one may expect that, for *N* → +∞, the quadratic term ${\tilde{\kappa }}_{3}$ is a good approximation for ${\bar{\kappa }}_{3}$. Indeed, Fig 5 illustrates this. Thus, we are able to conclude that, in the limit of large networks, the dominating contribution to the average joint third cumulant ${\bar{\kappa }}_{3}$ comes from the trees of topology *T*_6_ present in the network. One more thing to note is that the leading term ${\tilde{\kappa }}_{3}$ is proportional to a power of the stationary rate $\bar{\Lambda }$. Let us briefly consider what happens to $\bar{\Lambda }$ in very large networks, for *N* → +∞. We have

$$\bar{\Lambda }=\frac{1}{1-Np\left(\frac{N_{E}}{N}g_{E}+\frac{N_{I}}{N}g_{I}\right)}\to 0\text{,}\;N\to +\infty ,$$ (50)

assuming we keep all other parameters fixed. As a result of this, the product $N^{2}{\bar{\Lambda }}^{5}$ in ${\tilde{\kappa }}_{3}$, will decay to zero with increasing network size. Thus, when the size of the network considered grows without bounds, two things happen:

- The leading term ${\tilde{\kappa }}_{3}$ becomes a better approximation of the average third cumulant ${\bar{\kappa }}_{3}$ but, at the same time,
- The average third cumulant ${\bar{\kappa }}_{3}$ itself goes to 0.

The second point shouldn’t be too surprising. Indeed, once we remember that ${\bar{\kappa }}_{3}$ is proportional to the skewness of the population activity (defined as the sum of spike counts of all neurons in the network, in a bin of size *T*), its asymptotic vanishing is a straightforward consequence of the Central Limit Theorem. As *N* increases, the population activity is behaving more and more like a Gaussian random variable and, as a consequence, its skewness inevitably decays to zero. This effect is reflected by the horizontal asymptote in Fig 5.

### The signs of tree motif contributions to the third cumulant in regular networks depend on their topological structure

In this section, we will analyze the contributions of terms, corresponding to tree shapes in Fig 4 with fixed branch length. More precisely, let us consider once again Eq 25, plugging in the power series expansion of matrix *B* and exchanging the order of summation over “branch length” (i.e. summation over powers of the *G* matrix) and summation “over nodes” (i.e. summation over *i*, *j* and *k*, used to define ${\bar{\kappa }}_{3}$), we get

$$\begin{array}{ll} {\bar{\kappa }}_{3} & =\frac{\bar{\Lambda }}{N^{3}}\underset{l_{1},l_{2},l_{3}}{\sum }\left[\underset{i,j,k,m}{\sum }G_{im}^{l_{1}}G_{jm}^{l_{2}}G_{km}^{l_{3}}\right]\\ & +\frac{3\bar{\Lambda }}{N^{3}}\underset{l_{1},l_{2}}{\sum }\left[\underset{i,j,k}{\sum }G_{ik}^{l_{1}}G_{jk}^{l_{2}}\right]\\ & +\frac{3\bar{\Lambda }}{N^{3}}\underset{l_{1},l_{2},l_{3}}{\sum }\left[\underset{i,j,k,m}{\sum }G_{im}^{l_{1}}G_{jm}^{l_{2}}G_{mk}^{l_{3}}\right]\\ & \frac{6\bar{\Lambda }}{N^{3}}\underset{l_{1},l_{2},l_{3}}{\sum }\left[\underset{i,j,k,n}{\sum }G_{ij}^{l_{1}}G_{jn}^{l_{2}}G_{kn}^{l_{3}}\right]\\ & +\frac{6\bar{\Lambda }}{N^{3}}\underset{l_{1},l_{2}}{\sum }\left[\underset{i,j,k}{\sum }G_{ij}^{l_{1}}G_{jk}^{l_{2}}\right]\\ & +\frac{3\bar{\Lambda }}{N^{3}}\underset{l_{1},l_{2},l_{3},l_{4}}{\sum }\left[\underset{i,j,k,m,n}{\sum }G_{im}^{l_{1}}G_{jm}^{l_{2}}G_{mn}^{l_{4}}G_{kn}^{l_{3}}\right]. \end{array}$$ (51)

The terms in the square brackets can be interpreted as the total weight of all relevant trees (see Fig 4) present in the network, with lengths of all branches fixed. Under the regularity assumption, i.e. if all neurons have the same in-degree and out-degree, it is straightforward to conclude that the “square bracket term” of a tree T with *n* nodes and *l* leaves, embedded in a network of size *N*, can be computed as (see S2 Appendix)

$$N^{n}p^{l-1}\prod_{v}\mu^{\left(k_{v}\right)}{\left[\mu^{\left(1\right)}N\right]}^{l_{1}+\cdots +l_{n-1}-n+1},$$ (52)

where the product is over all internal nodes (i.e. nodes that are not leaves) of T and *k*_*v*_ is the out-degree of node *v*. The numbers *l*_1_, …, *l*_*n*−1_ encode the lengths of branches of T, of which there are exactly *n* − 1 in a tree with *n* nodes. In fact, it is this result that greatly simplifies the “summation over branch lengths” one needs to perform in order to obtain Eq 44.

Furthermore, from formula 52 we see that the only relevant characteristics of a tree T that determine the weight of the contribution are the number of its nodes *n*, the number of its leaves *l* and the out-degrees of its internal nodes. Note that the root counts as an internal node here. Trees with a large total branch length contribute relatively little to ${\bar{\kappa }}_{3}$. Indeed, as

$$|\mu^{\left(1\right)}N|=|p\left(N_{E}g_{e}+N_{I}g_{I}\right)|<1,$$ (53)

we have that, when the total length of all branches tends to infinity (i.e. when the sum *s*_*n*_ ≡ *l*_1_ + ⋯ + *l*_*n*−1_ grows beyond all bounds), the corresponding term ${\left(\mu^{\left(1\right)}N\right)}^{s_{n}}$ decays to zero.

Lastly, we consider the issue of determining the signs of various contributions to ${\bar{\kappa }}_{3}$. This can be done by once again closely analyzing formula 52. First, note that the common input terms *μ*^(*k*)^ are positive for even and negative for odd *k*. Indeed, as we assume that underlying network in inhibition-dominated (that is, if we assume that the total input to a neuron is negative) we have, in mathematical terms that

$$N_{E}g_{E}+N_{I}g_{I}<0\;\Leftrightarrow \;g_{I}<-\frac{N_{E}}{N_{I}}g_{E}.$$ (54)

Thus,

$$\begin{array}{ll} \mu^{\left(2r+1\right)} & =p\left(\frac{N_{E}}{N}g_{E}^{2r+1}+\frac{N_{I}}{N}g_{I}^{2r+1}\right)\\ & <p\left(\frac{N_{E}}{N}g_{E}^{2r+1}+{\left(-1\right)}^{2r+1}\frac{N_{E}}{N}{\left(\frac{N_{E}}{N_{I}}\right)}^{2r}g_{E}^{2r+1}\right). \end{array}$$

Therefore,

$$\mu^{\left(2r+1\right)}<p\frac{N_{E}}{N}\left[1-{\left(\frac{N_{E}}{N_{I}}\right)}^{2r}\right]g_{E}^{2r+1}<p\left[1-{\left(\frac{N_{E}}{N_{I}}\right)}^{2r}\right]g_{E}^{2r+1}<0,$$ (55)

since *g*_*E*_ > 0, *N*_*E*_ > *N*_*I*_ and 0 ≤ *p* ≤ 1. In the same way, one can show that *μ*^(2*r*)^ > 0. Therefore, the out-degree sequence of the internal nodes of the tree affects the sign of the corresponding contribution. If, for example, the tree has two internal nodes, with out-degrees 1 and 2, respectively, this will contribute an overall negative sign to the term. However, the out-degree sequence alone does not completely determine the sign of the contribution. Another factor is the parity of the total length of all branches, i.e. the sum *s*_*n*_ ≡ *l*_1_ + … + *l*_*n*−1_. To see why, note that *Nμ*^(1)^ < 0, by our previous discussion, and likewise

$${\left[\mu^{\left(1\right)}N\right]}^{s_{n}-n+1}\;$$ (56)

is either negative or positive, depending on whether *s*_*n*_ = 2*r* + 1 or *s*_*n*_ = 2*r*. (Note that *s*_*n*_ ≥ *n* − 1.)

**Fig 6:**
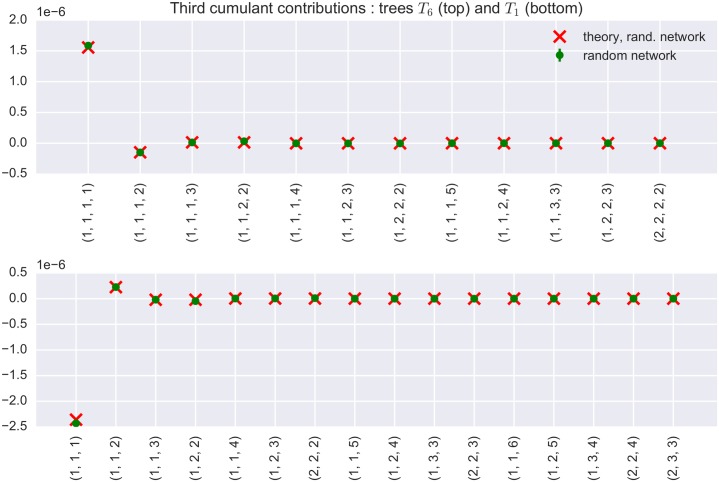
Contributions of some tree structures to the average third cumulant in a random network. Top: Theoretical (narrow out-degree distribution approximation) and sample contributions to the average third cumulant of *T*_6_ (see Fig 4) tree topologies with fixed branch lengths. Ticks on the x-axis code for the lengths of the four branches of the tree. The ordering of the indices in the tick labels is done in a top-to-bottom and left-to-right fashion. More precisely, the first number corresponds to the length of the “leftmost” branch emanating from the root node, and so forth. The sample contributions were computed as averages of 3 independent realizations of a random network. Bottom: Theoretical and sample contributions to the average third cumulant of *T*_1_ tree topologies with fixed branch lengths.

To summarize, the resulting sign of the total contribution to the average third cumulant, of a specific tree with *n* nodes, *l* leaves, a given out-degree sequence and branch lengths depends on both the parity of the product of the internal node out-degrees and the parity of the total branch length. What this means in practice is that the presence of certain trees increases the overall level of third order correlation, while the existence of others can actually have the opposite effect. Whether the latter or the former is the case depends solely on the tree’s topological structure, i.e. how the internal nodes branch and how many edges it contains. As an illustration, the signs and sizes of contributions of two sample trees in a recurrent random network are depicted in Fig 6. One can clearly see which trees increase third-order correlations in the network, and which trees actually decrease them.

One last thing to note is how quickly the contributions, involving higher matrix powers of *G* (i.e. those trees with higher total branch length) decay to zero as the total branch length increases. This behavior is essentially governed by the spectral radius *ρ*(*G*) of the connectivity matrix. For example, in a large random network of both excitatory and inhibitory neurons, the spectrum consists of a single eigenvalue of size $N\mu^{\left(1\right)}=Np(\frac{N_{E}}{N}g_{E}+\frac{N_{I}}{N}g_{I})$ and a bulk spectrum, contained within a circle in the complex plane [34]. Its radius *r* is asympotically given by

$$r^{2}=Np\left(1-p\right)\left(\frac{N_{E}}{N}g_{E}^{2}+\frac{N_{I}}{N}g_{I}^{2}\right).$$ (57)

While, as was already mentioned, the quantity *Nμ*^(1)^ corresponds to the total average input to a neuron, the radius *r* of the circle encompassing the bulk spectrum corresponds to the variance of this input. Thus, if the variance of the total input to a neuron in a random network is not too big (*r* < 1), it will exhibit the aforementioned decay of contributions from trees with higher total branch lengths.

### Excitatory hubs in the network increase third-order cumulants

In the previous sections, we have demonstrated that the average third cumulant in networks with narrow degree distributions is determined by global parameters such as the number of neurons *N*, the connection probability *p*, and the average strength of input shared by *k* neurons, *μ*^(*k*)^. Of course, in networks with a wide degree distribution, the regular network approximation (which we used to derive the equation in S2 Appendix) is no longer valid. To demonstrate some of the new phenomena by simulation, we consider a network model with a geometric degree distribution, originally introduced in [3]. In short, the out-degrees *k* of excitatory and inhibitory neurons are chosen from a geometric distribution with parameter *k*_0_ (representing the mean out-degree) according to

$$P\left(k\right)={\left(1-\frac{1}{k_{0}}\right)}^{k-1}\frac{1}{k_{0}}.$$ (58)

**Fig 7:**
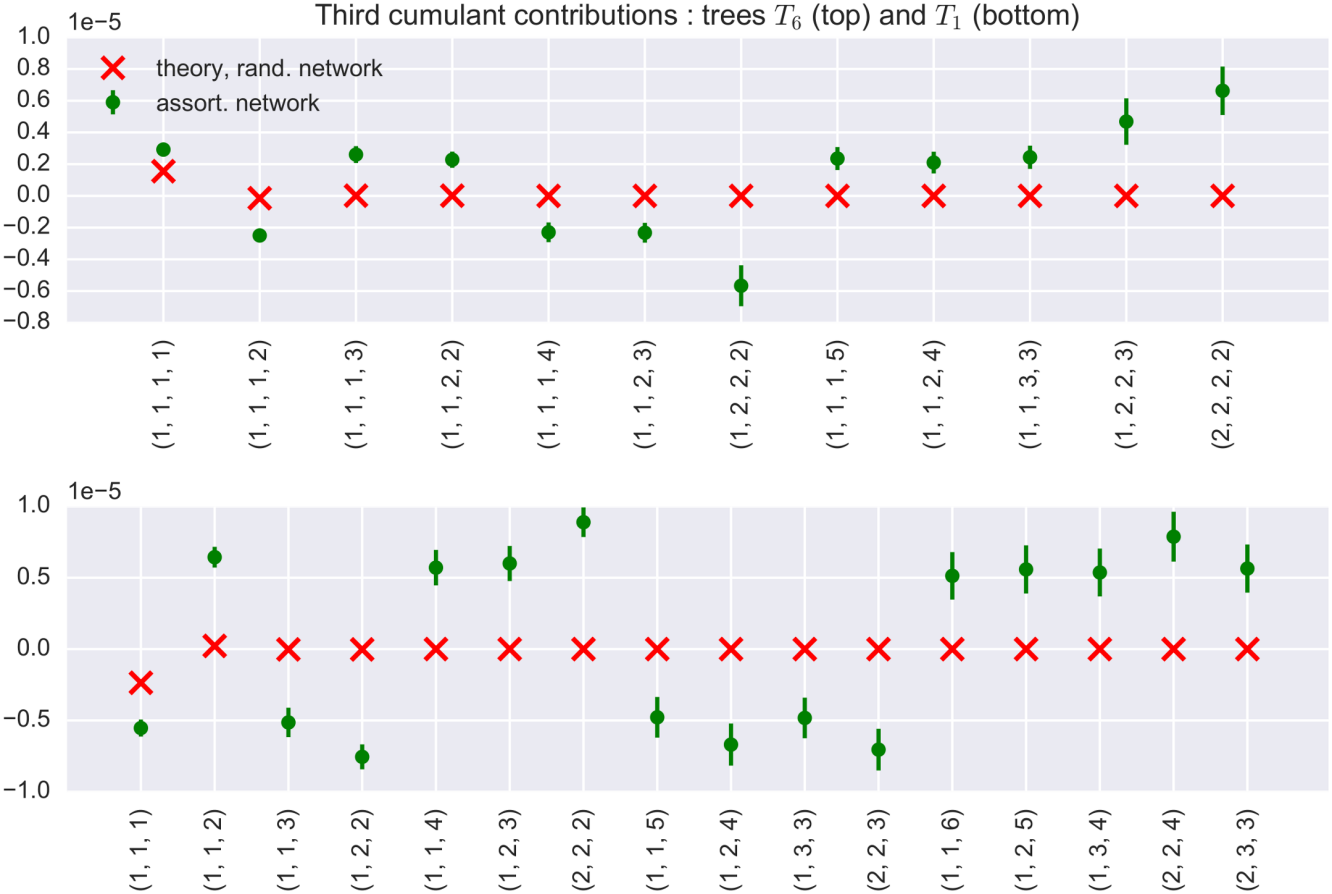
Contribution of tree motifs with longer total branch length increases in networks with excitatory hubs. Top: Theoretical (narrow out-degree distribution approximation for random networks) and sample contributions (in non-regular networks) of the average third cumulant of *T*_6_ (see Fig 4) tree topologies with fixed branch lengths. Ticks on the x-axis code for the lengths of the branches of the tree. The ordering of the indices in the tick labels is done in a top-to-bottom and left-to-right fashion. The sample contributions were computed as averages of 3 independent realizations of an assortative network with a geometric out-degree distribution. Bottom: Theoretical and sample contributions to the average third cumulant of *T*_1_ tree topologies with fixed branch lengths.

This distribution exhibits a mean connection probability of 1/*k*_0_ and a long tail. After the sampling of out-degrees, excitatory neurons are divided into “hubs” (out-degree *k* > *k*_0_) and “non-hubs” (*k* ≤ *k*_0_). Postsynaptic neurons for non-hubs and inhibitory neurons are chosen randomly from the population consisting of all other neurons. However, for hub neurons, a fixed fraction *f* of all outgoing connections goes to other hubs. By varying *f* between 0 and 1, one can choose how densely connected the subnetwork of hubs will be. The “critical value” to keep in mind here is *f*_0_ = 0.35. If *f* > *f*_0_, hub neurons have a preference to connect to other hubs. Such a network is called “assortative”, otherwise it is called “disassortative”, see [3] for details. Similar networks have been studied in [35, 36]. The effect of the geometric out-degree distribution on the distribution of network motifs is depicted in Fig 7.

If excitatory hubs preferentially connect to other hubs (for assortative networks), the number of relevant tree motifs with high total branch length grows in the network, and so does their combined strength. This is one major difference between assortative and random networks, and a reason why the contributions of longer trees in networks with hubs tend to be much larger than in Erdős-Rényi topologies. Of course, along the same lines, the number of “short” motifs (i.e. those with small total branch length) decreases (in comparison to their “longer” counterparts). This phenomenon is illustrated in Fig 7.

This discrepancy can also be used to say something about the topology of the network that generated a given set of recorded spike data. Indeed, once the connection probability and third order correlations have been estimated (e.g. with the help of k-statistics), one could compare the regular network theory predictions with the third order cumulants obtained from data. A large disparity between the two could imply, for example, the presence of hubs and a wide in- and out-degree distribution in the network that generated the data.

## Discussion

In this work, we have studied connections between topology and measures of average third-order correlations in networks of spiking neurons. We have compared different connectivity rules with respect to their effect on the average joint third cumulant, ${\bar{\kappa }}_{3}$. Furthermore, we showed which topological motifs in the network contribute to the overall strength of third-order correlations. While our focus was on network models arising in neuroscience, we feel that the results presented here could as well be relevant in other fields, where correlations of higher-order play an important role.

As a handy computational model of spiking neuronal activity, we have used the Hawkes point process [27, 28], which was originally introduced as a model of earthquake activity. It is sufficiently rich in order to model interesting dependencies between various types of events (in our case, spikes of different neurons), but still simple enough to be tractable. Indeed, these are the exact properties that make Hawkes processes quite useful models in neuroscience. They have been employed in the analysis of pairwise correlations between spike trains [3, 37], modeling spike-timing dependent plasticity [38, 39], and, very recently, to model single unit activity recorded on a monkey during a sensory-motor task [40].

Using the Hawkes process theory, we have shown that a linear stochastic point process model can reproduce not only the event rates and pairwise correlations in networks (as was already shown in [3]), but also its third-order joint cumulants, which are statistical measures of correlations between groups of three nodes. These cumulants can be seen as a quantification of “non-Gaussian” properties of the total population activity observed in time bins of a given size.

The problem of quantifying higher-order correlations is of some importance in computational neuroscience. It has been suggested a long time ago [41, 42] that understanding the cooperative dynamics of populations of neurons would provide much needed insight into the neuron-level mechanisms of brain function. Indeed, there is now a large body of experimental evidence that supports the idea of computationally relevant correlations between neurons in a network [7, 43–45]. The evidence for coordinated activity of neuronal spike trains, however, mostly relies on the correlations between pairs of nerve cells [46–50]. Unfortunately, it is becoming increasingly clear that pairwise correlations cannot explain the intricate dynamics of neuronal populations [9, 12, 51, 52] and that higher-order moments of spiking activity need to be taken into account.

Traditionally in neuroscience, higher-order synchrony has been almost exclusively investigated with the help of classical tools borrowed from statistical physics such as maximum entropy models [13–18, 53]. In this approach, the quantifiers of higher-order coordination are the so-called “interaction parameters” of the binary exponential family. However, an alternative measure, commonly used in statistical literature, also exists—it is the joint cumulant. As already mentioned in [54, 55], cumulant correlations are not identical to the higher order exponential family parameters (for details, see [54]). In a sense, it can be said that non-zero cumulants indicate the presence of additive common input (a well-known model for correlated stochastic signals, see [56–58]), while the interaction parameters of maximum entropy models measure multiplicative interactions. The mathematical differences between the two types of dependence are currently under investigation [59–61]. As our neuronal network model each neuron “feels” only the linear sum of spiking activity of its presynaptic partners, in this work we have opted for quantifying synchrony using joint cumulants. Finally, it may be worthwhile to note that there are other ways of generating time structured correlations of higher order in computational models (see, for example, [9], but also [62]).

In addition, by generalizing the result in [3], we have found that integrated third-order correlations (*κ*^*ijk*^) also admit a representation in terms of sums of weights of certain topological sub-motifs in the network. While in the case of pairwise correlations between neurons these motifs were simple binary trees (see Fig 2), when dealing with third-order interactions the motifs become more complex (Fig 3) “trees with three leaves”, which are still manageable computationally. More precisely, it is the combined “strength” of all such trees containing a triplet of neurons that determine how often, on average, the activity of such a triplet exhibits coordinated spiking. Sadly, no concise matrix product formula is available for the whole third cumulant tensor {*κ*^*ijk*^}_*i*, *j*, *k*_ and one has to resort to writing down equations for individual components, which still offer the possibility of efficient estimation. Indeed, computing the theoretical cumulants *κ*^*ijk*^ for (close to) regular networks is much less computationally intensive than estimating them from data via *k*-statistics and only relies on simple algebraic manipulations of the connectivity matrix *G*.

We have also studied analytically the average third-order cumulant ${\bar{\kappa }}_{3}$, derived from the sum of joint cumulants of all possible triplets of neurons in the network. We have shown that the value of ${\bar{\kappa }}_{3}$ in random networks of Erdős-Rényi type does not depend on fine-scale topological structure and is instead a function of global network parameters, such as the network size *N*, the connection probability *p* and total common input to groups of neurons. Furthermore, we have shown that, in the limit of very large networks, the dominating contribution to ${\bar{\kappa }}_{3}$ comes from the combined weight of all trees with a specific topology (which we denoted *T*_6_, see Fig 4) present in the network. Thus, for large, random networks, it is tree-like connectivity motifs of this topology that affects the average third cumulant most.

We were able to show that the contributions of individual subtrees to the average joint cumulant depend on specific topological properties of the tree, such as its number of branches, number of nodes and, interestingly, the out-degrees of its internal nodes (nodes that are not leaves as they have a nonzero out-degree). Not surprisingly, in a stable network (whose connectivity matrix *G* has a spectral radius less than 1), the absolute contributions of trees with a large number of branches decays to 0 as the number of branches increases. However, the sign of the total contribution turns out to depend both on the parity of the sum of all internal node out-degrees and the parity of the total branch length. This, in principle, allows one to determine whether the presence of a particular sub-tree in a network will increase or decrease the third cumulant, and thus allow to compute the total size of third-order interactions.

Finally, we considered a case in which our regular network approximation fails, networks with interconnected hub neurons. Similar networks were already considered in [3]. Their main characteristic is a heavy-tailed out-degree distribution (in the case we considered, it was geometric). Such networks are, in a sense, the opposite of an Erdős-Rényi type random network. The presence of interconnected hubs increases the number of subtrees in the network with large total branch length and, consequently, their overall contribution to the average joint third cumulant. Thus, such networks illustrate nicely how “higher-order” motifs can, for certain networks, influence the overall third-order cumulant structure, which is not possible in networks with narrow out-degree distributions.

As far as the limitations of our approach are concerned, it is important to note that the linear theory of Hawkes processes which we resorted to [29] is strictly valid only for purely excitatory networks, as the instantaneous rate function is not allowed to become negative. For the case discussed here, this may happen, as the networks are inhibition-dominated. However, in accordance with what was already mentioned in [27], the theoretical results remain approximately valid for networks with negative interactions, as long as the probability of the rate being negative is small. Still, an interesting generalization of our model, and the results achieved with it, would be the case of multiplicative interaction [63]. More generally, a point process model in which an non-negative nonlinearity is applied to Eq 3 yields a necessarily positive rate for any choice of interaction kernels. The computational approach one would have to use in this case in order to study the effect of topology on higher-order correlations would be quite different, though, as almost no analytical results exist for such models [64, 65].

## Supporting Information

S1 Appendix Integrated covariances and joint third cumulants. (PDF)

S2 AppendixComputation of ${\bar{\kappa }}_{3}$.(PDF)

S3 Appendix Derivation of the third-order joint cumulant formula. (PDF)
