# Supplementary material for: Interplay between Graph Topology and Correlations of Third Order in Spiking Neuronal Networks
Source: PLoS Comput Biol. 2016 Jun 6;12(6):e1004963. doi: 10.1371/journal.pcbi.1004963 (PMC4894630; doi:10.1371/journal.pcbi.1004963)
Supplement: S1 Appendix — (PDF) [file pcbi.1004963.s001.pdf]

# Integrated covariances and joint third cumulants

In this section, we prove that, for a stationary point process,

$$C_{ij} = \lim_{T\to+\infty} \frac{\mathrm{cov}[N_i(T), N_j(T)]}{T}, \tag{1}$$

and

$$\kappa^{ijk} = \lim_{T\to+\infty} \frac{\kappa_3(N_i(T), N_j(T), N_k(T))}{T}. \tag{2}$$

To prove the first identity, we proceed as follows. Note that

$$\mathrm{cov}[N_i(T), N_j(T)] = \int_0^T \int_0^T \mathrm{cov}[dN^i_{t_1}, dN^j_{t_2}] dt_1 dt_2. \tag{3}$$

Next, we let $C_{ij}(t_1, t_2) \equiv \mathrm{cov}[dN_i(t_1), dN_j(t_2)]$ be the non-stationary covariance density, measuring the probability of non-chance occurrence of a spike pattern $\{(i, t_1), (j, t_2)\}$. For a stationary process, we have that

$$C_{ij}(t_1, t_2) = C_{ij}(t_2 - t_1), \tag{4}$$

i.e. it is a function of only the difference between spike times $t_1$ and $t_2$. Thus,

$$\int_0^T \int_0^T C_{ij}(t_1, t_2) dt_1 dt_2 = \int_0^T \int_0^T C_{ij}(t_2 - t_1) dt_1 dt_2. \tag{5}$$

Now, introducing a change of variables $\tau \equiv t_2 - t_1$, we get

$$\int_0^T \int_0^T C_{ij}(t_2 - t_1) dt_1 dt_2 = \int_{-T}^T C_{ij}(\tau) \left( \int_{-\tau}^{T-\tau} dt_1 \right) d\tau = T \int_{-T}^T C_{ij}(\tau) d\tau, \tag{6}$$

as the inner integral equals $T$. We have obtained

$$\mathrm{cov}[N_i(T), N_j(T)] = T \int_{-T}^T C_{ij}(\tau) d\tau \Leftrightarrow \frac{\mathrm{cov}[N_i(T), N_j(T)]}{T} = \int_{-T}^T C_{ij}(\tau) d\tau. \tag{7}$$

Now, letting $T \to +\infty$, we get

$$C_{ij} = \lim_{T\to+\infty} \frac{\mathrm{cov}[N_i(T), N_j(T)]}{T}. \tag{8}$$

The proof is analogous in the three-dimensional case, from which we conclude that

$$\kappa^{ijk} = \lim_{T\to+\infty} \frac{\kappa_3[N_i(T), N_j(T), N_k(T)]}{T}. \tag{9}$$
