# Supplementary material for: Interplay between Graph Topology and Correlations of Third Order in Spiking Neuronal Networks
Source: PLoS Comput Biol. 2016 Jun 6;12(6):e1004963. doi: 10.1371/journal.pcbi.1004963 (PMC4894630; doi:10.1371/journal.pcbi.1004963)
Supplement: S2 Appendix — (PDF) [file pcbi.1004963.s002.pdf]

Computation of $\bar{\kappa}_3$

In this section, we derive the regular graph approximation for the average joint third cumulant. We start with the following formula.

$$\begin{aligned}\bar{\kappa}_3 = & \frac{\bar{\Lambda}}{N^3}\sum_{l_1,l_2,l_3}\left[\sum_{i,j,k,m} G^{l_1}_{im}G^{l_2}_{jm}G^{l_3}_{km}\right] \\ & + \frac{3\bar{\Lambda}}{N^3}\sum_{l_1,l_2}\left[\sum_{i,j,k} G^{l_1}_{ik}G^{l_2}_{jk}\right] \\ & + \frac{3\bar{\Lambda}}{N^3}\sum_{l_1,l_2,l_3}\left[\sum_{i,j,k,m} G^{l_1}_{im}G^{l_2}_{jm}G^{l_3}_{mk}\right] \\ & + \frac{6\bar{\Lambda}}{N^3}\sum_{l_1,l_2,l_3}\left[\sum_{i,j,k,n} G^{l_1}_{ij}G^{l_2}_{jn}G^{l_3}_{kn}\right] \\ & + \frac{6\bar{\Lambda}}{N^3}\sum_{l_1,l_2}\left[\sum_{i,j,k} G^{l_1}_{ij}G^{l_2}_{jk}\right] \\ & + \frac{3\bar{\Lambda}}{N^3}\sum_{l_1,l_2,l_3,l_4}\left[\sum_{i,j,k,m,n} G^{l_1}_{im}G^{l_2}_{jm}G^{l_4}_{mn}G^{l_3}_{kn}\right].\end{aligned}$$

These 6 terms each correspond to one of the 6 possible rooted trees, depicted in Fig. 4. First, let us compute the terms inside the square brackets. The following result holds :

Let $\mathcal{T}$ be an arbitrary rooted tree with at most N nodes. Then, the "square bracket" corresponding to it (obtained by summing over all possible indices of nodes in a tree with a fixed number of branches) can be computed using the following pseudo-algorithm :

1. *Set $X = 1$.*
2. *For every leaf of $\mathcal{T}$, $X \leftarrow X * N$*
3. *For every edge in $\mathcal{T}$, $X \leftarrow X * p$*
4. *For every internal node of out-degree k (where the root counts as an internal node) of $\mathcal{T}$, $X \leftarrow X * \frac{\mu^{(k)} \cdot N}{p}$, where $\mu^{(k)}$ is defined as the average common input, shared by k nodes, and is defined by*

$$\mu^{(k)} = p\sum_t \frac{N_t}{N} g_t^k. \tag{1}$$

It is not difficult to prove that this is equivalent to :

Let $\mathcal{T}$ be an arbitrary rooted tree with $n \le N$ nodes and $l \le n-1$ leaves. Then, the corresponding "square bracket term" is equal to

$$N^n p^{l-1} \prod_v \mu^{(k_v)} \left[\mu^{(1)} N\right]^{l_1+\cdots+l_{n-1}-n+1}, \tag{2}$$

where the product is over all internal nodes (i.e. nodes that are not leaves) of $\mathcal{T}$ and k_v is the out-degree of node v. The numbers $l_1, \cdots, l_{n-1}$ encode the lengths of branches of $\mathcal{T}$, of which there are $n-1$ (in a tree with n nodes).

We now use the previous result to compute "square bracket terms" for all the trees in Fig. 4. We have

1. T_1 :

$$X_1 = N^4 p^2 \mu^{(3)} \left[\mu^{(1)} N\right]^{l_1+l_2+l_3-3},$$

   where l_1, l_2 and l_3 are fixed lengths of the three branches of the first tree and $l_1, l_2, l_3 \ge 1$.

2. T_2 :

$$X_2 = N^3 p \mu^{(2)} \left[\mu^{(1)} N\right]^{l_1+l_2-2}.$$

3. T_3, T_4 - they have the same number of nodes and their internal nodes have the same distribution of out-degrees :

$$X_3, X_4 = N^4 p \mu^{(1)} \mu^{(2)} \left[\mu^{(1)} N\right]^{l_1+l_2+l_3-3}.$$

4. T_5 :

$$X_5 = N^3 \left[\mu^{(1)}\right]^2 \left[\mu^{(1)} N\right]^{l_1+l_2-2}.$$

5. T_6 :

$$X_6 = N^5 p^2 \left[\mu^{(2)}\right]^2 \left[\mu^{(1)} N\right]^{l_1+l_2+l_3+l_4-4}.$$

Now, we are finally ready to compute the sums of the "bracket terms" over the lengths of branches $\{l_i\}_{1\le i\le k}$. Looking at the expressions for $\{X_k\}_{1\le k\le 6}$, it is easy to see that the only place where the lengths l_i explicitly appear is as powers of the term $\mu^{(1)}N$. Thus, to compute sums over lengths of branches, we have to be able to compute

$$S_k \equiv \sum_{l_1,l_2,\cdots,l_k} a^{\sum_{i=1}^{k}(l_i-1)}. \tag{3}$$

First, we make the following observation.

The sum S_k can be written as

$$S_k = \sum_{r\ge k} \binom{r-1}{k-1} a^{r-k} = \sum_{r\ge k} \frac{1}{(k-1)!}(r-1)(r-2)\cdots(r-k+1)a^{r-k} \tag{4}$$

To see why this is true, consider that, by definition, $S_k = \sum_{l_1} \sum_{l_2} \cdots \sum_{l_k} a^{\sum_{i=1}^{k}(l_1-1)}$. *If we introduce a new variable* $r = \sum_{i=1}^{k} l_i$*, we have that* $r \geq k$*, as* $l_i \geq 1$*, for all* i*. Furthermore, the number of different* k*-tuples* $(l_1, \cdots, l_k)$ *that result in the same value of* r *is equal to the number of compositions of the number* r *into* k *parts, which is equal to* $\binom{r-1}{k-1}$*. The claim then readily follows.*

In addition, we also have the following :

The sum S_k *is explicitly summable for all* $|a| < 1$ *and equals*

$$S_k = \frac{(-1)^{k-2}}{(1-a)^k}. \tag{5}$$

To prove this fact, we start from the result of the previous proposition and substitute $r - 1 = x$*, getting*

$$S_k = \sum_{x \geq k-1} \frac{1}{(k-1)!} x(x-1)\cdots(x-k+2)a^{x-(k-1)}. \tag{6}$$

Now, letting $n = k - 1$ *and* $r = x$ *we obtained*

$$S_{n+1} = \frac{1}{n!} \sum_{r \geq n} r(r-1)\cdots(r-n+1)a^{r-n}. \tag{7}$$

On the other hand, for $|a| < 1$*,*

$$\begin{aligned} \frac{d}{da^n}\left(\frac{1}{1-a}\right) &= \frac{d}{da^n}\sum_{r\geq 0} a^r = \sum_{r\geq 0} r(r-1)\cdots(r-n+1)a^{r-n} \\ &= \sum_{r\geq n} r(r-1)\cdots(r-n+1)a^{r-n}, \end{aligned}$$

and therefore

$$S_{n+1} = \frac{1}{n!}\frac{d}{da^n}\left(\frac{1}{1-a}\right). \tag{8}$$

But, by induction,

$$\frac{d}{da^n}\left(\frac{1}{1-a}\right) = \frac{(-1)^{n-1}n!}{(1-a)^{n+1}}. \tag{9}$$

Thus, we have, after letting $n + 1 = k$*,*

$$S_k = \frac{(-1)^{k-2}}{(1-a)^k}, \tag{10}$$

which proves our claim.

We are now almost done. Indeed, from our previous considerations, we have

1. T_1 ($k = 3$, $S_3 = \frac{-1}{(1-a)^3}$):

$$\sum_{l_1,l_2,l_3} N^4 p^2 \mu^{(3)} \left[\mu^{(1)} N\right]^{l_1+l_2+l_3-3} = \frac{-N^4 p^2 \mu^{(3)}}{(1-\mu^{(1)}N)^3} \tag{11}$$

2. T_2 $(k=2,\, S_2 = \frac{1}{(1-a)^2})$:

$$\sum_{l_1,l_2} N^3 p\mu^{(2)} \left[\mu^{(1)} N\right]^{l_1+l_2-2} = \frac{N^3 p\mu^{(2)}}{(1-\mu^{(1)}N)^2} \tag{12}$$

3. T_3, T_4 $(k=3)$:

$$\sum_{l_1,l_2,l_3} N^4 p\mu^{(1)}\mu^{(2)} \left[\mu^{(1)} N\right]^{l_1+l_2+l_3-3} = \frac{-N^4 p\mu^{(1)}\mu^{(2)}}{(1-\mu^{(1)}N)^3} \tag{13}$$

4. T_5 $(k=2)$:

$$\sum_{l_1,l_2} N^3 \left[\mu^{(1)}\right]^2 \left[\mu^{(1)} N\right]^{l_1+l_2-2} = \frac{N^3 \left[\mu^{(1)}\right]^2}{(1-\mu^{(1)}N)^2} \tag{14}$$

5. T_6 $(k=4,\, S_4 = \frac{1}{(1-a)^4})$:

$$\sum_{l_1,l_2,l_3,l_4} N^5 p^3 \left[\mu^{(2)}\right]^2 \left[\mu^{(1)} N\right]^{l_1+l_2+l_3+l_4-4} = \frac{N^5 p^3 \left[\mu^{(2)}\right]^2}{(1-\mu^{(1)}N)^4} \tag{15}$$

Finally, we are ready to write the formula for $\bar{\kappa}_3$. It reads

$$\begin{aligned}\bar{\kappa}_3 = {} & \frac{\bar{\Lambda}}{N^3}\frac{-N^4 p^2 \mu^{(3)}}{(1-\mu^{(1)}N)^3} + \frac{3\bar{\Lambda}}{N^3}\frac{N^3 p\mu^{(2)}}{(1-\mu^{(1)}N)^2} + \frac{3\bar{\Lambda}}{N^3}\frac{-N^4 p\mu^{(1)}\mu^{(2)}}{(1-\mu^{(1)}N)^3} \\ & + \frac{6\bar{\Lambda}}{N^3}\frac{-N^4 p\mu^{(1)}\mu^{(2)}}{(1-\mu^{(1)}N)^3} + \frac{6\bar{\Lambda}}{N^3}\frac{N^3 \left[\mu^{(1)}\right]^2}{(1-\mu^{(1)}N)^2} + \frac{3\bar{\Lambda}}{N^3}\frac{N^5 p^3 \left[\mu^{(2)}\right]^2}{(1-\mu^{(1)}N)^4}.\end{aligned}$$
