# Supplementary material for: Interplay between Graph Topology and Correlations of Third Order in Spiking Neuronal Networks
Source: PLoS Comput Biol. 2016 Jun 6;12(6):e1004963. doi: 10.1371/journal.pcbi.1004963 (PMC4894630; doi:10.1371/journal.pcbi.1004963)
Supplement: S3 Appendix — (PDF) [file pcbi.1004963.s003.pdf]

Measuring dependence between 2 or more variables

A core notion in probability theory and statistics is the covariance of two random variables X and Y, often denoted by $\mathrm{cov}[X, Y]$. Intuitively, it represents a measure of statistical dependence between X and Y and is computed as

$$\mathrm{cov}[X, Y] \equiv \mathbb{E}[XY] - \mathbb{E}[X]\mathbb{E}[Y]. \tag{1}$$

To clarify *why* the covariance is, in fact, an intuitive way of quantifying pairwise dependence, let us look at the special case of binary random variables, i.e. assuming X and Y can only take values 0 or 1. Using the definition of covariance and properties of binary random variables, we find that the covariance equals

$$P\{X \text{ and } Y \text{ are both equal to } 1\} - P\{X \text{ is equal to } 1\}P\{Y \text{ is equal to } 1\}.$$

Thus, the covariance $\mathrm{cov}[X, Y]$ is the probability of both X and Y having the a value of 1 at the same time, minus the probability that this happens due to a random coincidence. Indeed, assuming that X and Y are statistically independent, the probability of both being equal to 1 is exactly

$$P\{X \text{ is equal to } 1\}P\{Y \text{ is equal to } 1\}. \tag{2}$$

Now, let us generalize this concept further and ask the question: How can we measure the inter-dependence of three random variables X, Y and Z? For simplicity, let us assume that they, too, are binary. The naive thing to try is to define the "third-order covariance" $\kappa_3[X, Y, Z]$ as

$$P\{X, Y \text{ and } Z \text{ all equal } 1\} - P\{X \text{ equals } 1\}P\{Y \text{ equals } 1\}P\{Z \text{ equals } 1\}, \tag{3}$$

or, in terms of expectations,

$$\mathbb{E}[XYZ] - \mathbb{E}[X]\mathbb{E}[Y]\mathbb{E}[Z]. \tag{4}$$

At first sight, this seems decent enough. We measure the third-order dependence by taking all the cases in which the three variables have the same value, and then subtract the cases in which this is only due to pure chance. But we are missing something crucial. What if only X and Y are truly dependent? What if, indeed, $\mathrm{cov}[X, Y] \neq 0$, but the fact that Z has the same value is simply due to chance?

To account for this case, we have to subtract $\mathrm{cov}[X, Y]P\{Z = 1\}$ from our naive formula for the third-order covariance. However, since there is nothing special about the variable Z, we must also subtract $\mathrm{cov}[X, Z]P\{Y = 1\}$ and $\mathrm{cov}[Y, Z]P\{X = 1\}$ to account for the other two, complementary cases. Therefore, the full formula for $\kappa_3[X, Y, Z]$ must read

$$\begin{aligned}\kappa_3[X, Y, Z] = \mathbb{E}[XYZ] \\ &- \mathrm{cov}[X, Y]\mathbb{E}[Z] - \mathrm{cov}[X, Z]\mathbb{E}[Y] - \mathrm{cov}[Y, Z]\mathbb{E}[X] \\ &- \mathbb{E}[X]\mathbb{E}[Y]\mathbb{E}[Z].\end{aligned}$$

Plugging in Eq. 1, we get

$$\kappa_3[X,Y,Z] = \mathbb{E}[XYZ] - \mathbb{E}[XY]\mathbb{E}[Z] - \mathbb{E}[XZ]\mathbb{E}[Y] - \mathbb{E}[YZ]\mathbb{E}[X] + 2\mathbb{E}[X]\mathbb{E}[Y]\mathbb{E}[Z], \tag{5}$$

which is the definition of joint third cumulant of variables X, Y and Z in terms of their first and second mixed moments. The third cumulant measures the inter-dependence of three random variables in the same way that covariance does for two. In fact, covariance is nothing more than the cumulant of second order.

Of course, cumulants of orders $n > 3$ can also be defined, and their definitions and interpretations are analogous. Indeed, we can consider now an arbitrary n-dimensional random vector $\mathbf{X} = (X_1, \cdots, X_n) \equiv X_{\bar{n}}$, where we used the symbol $\bar{n}$ to denote the set $\{1, \cdots, n\}$. The cumulant of order n, denoted by $\kappa[X_{\bar{n}}]$, is a general measure of statistical dependence of the components of $\mathbf{X}$. It is defined, combinatorially, as

$$\kappa[X_{\bar{n}}] = \sum_{\pi} (|\pi| - 1)!(-1)^{|\pi|-1} \prod_{B\in\pi} \mathbb{E}\left[X_B\right], \tag{6}$$

where the sum goes over all partitions π of the set $\{1, \cdots, n\}$, $|\cdot|$ denotes the number of blocks of a given partition, and

$$\mathbb{E}[X_B] = \mathbb{E}\left[\prod_{i\in B} X_i\right] \tag{7}$$

is the mixed moment of all those components of $\mathbf{X}$, whose indices are in B. A dual formula, expressing moments in terms of cumulants, reads

$$\mathbb{E}[X_{\bar{n}}] = \sum_{\pi} \prod_{B\in\pi} \kappa[X_B], \tag{8}$$

where $\kappa[X_B]$ denotes the cumulant of those components of $\mathbf{X}$, whose indices are in B.
